# Understanding Iron Impurities in Australian Kaolin and Their Effect on Acid and Heat Activation Processes of Clay

**DOI:** 10.1021/acsomega.2c06795

**Published:** 2023-02-06

**Authors:** Bhabananda Biswas, Md. Rashidul Islam, Amal Kanti Deb, Anthony Greenaway, Laurence N. Warr, Ravi Naidu

**Affiliations:** † Global Centre for Environmental Remediation, The University of Newcastle, Callaghan, NSW 2308, Australia; ‡ crcCARE Pty Ltd., ATC Building, The University of Newcastle, Callaghan, NSW 2308, Australia; § Latin Resources Ltd., Unit 3, 32 Harrogate Street, West Leederville, WA 6007, Australia; ∥ Institute of Geography and Geology, University of Greifswald, Greifswald 17489, Germany

## Abstract

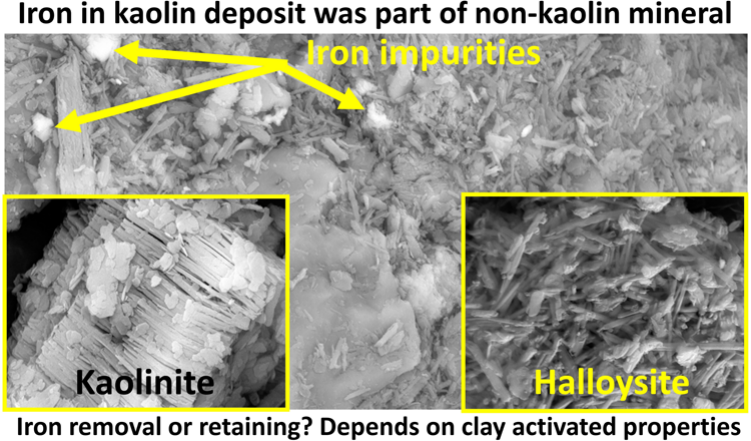

Iron impurities present in the crystal structure of kaolin minerals or in accessory species are frequently encountered in clay deposits. As knowledge of the location and states of the iron is crucial when modifying the properties of clays by activation, it is important that new deposits are well characterized in terms of the amount and location of this metal. The Western Australia Noombenberry deposit has been identified as a large resource of kaolin composed largely of halloysite and kaolinite. We sampled six from one hundred drill holes and grouped them according to major mineral and iron impurities. First, we characterized them to understand the source of iron impurities. Then, we performed three physicochemical activation processes of samples involving acid treatment (by 3 M HCl), heating at 600 °C, and a combination of both. State-of-the-art tools, including X-ray diffraction, X-ray photoelectron spectroscopy, scanning and transmission electron microscopy, and nuclear magnetic resonance, revealed the properties of kaolin, iron impurities, and the changes incurred after activation. The iron impurities were found to be linked to non-kaolin minerals, i.e., in mica or illite. Once the iron was removed mainly by acid activation, the surface area, pore volume, and negative surface charges increased, and that was significant for halloysite-rich samples. These properties helped adsorb N_2_ gas compared to the raw kaolin. Therefore, knowing the iron’s location and states in associated mineral species and their dissolution/retention may expand the scope of material development for gas adsorption. They are also useful in other applications like clay purification and adsorbent or additive formulations.

## 1 Introduction

Deposits of kaolin clay containing mixtures of kaolinite (Kln) and halloysite (Hly) are known to be of economic value, particularly when halloysite is the dominant mineral.^1,2^ For improved material properties, pristine clays may require activation for certain purposes, such as thermal or acid treatment or a combination of both.^3^ Other modifications of these activated materials are used to synthesize clay nanocomposites suitable for advanced applications involving sorption, supports for catalysts or microorganisms, and additives for fertilizers or feeds.^2−5^ The key physicochemical properties that are commonly modified include the crystallinity, surface net charge, surface area, or pore volume.^6^ The presence of iron (Fe) oxides in clay mineral assemblages is known to significantly influence the activation outcomes during preparation.^7^ Key causes of achieving modified properties of clays are the Fe and redox changes. Iron impurities can be coexisting oxides, mica/illite associates, or both.^7^ Understanding these aspects of clay from a newly identified deposit is of great importance toward valorization of clays. For instance, taking a deposit in Vrbica, Arandjelovac basin, Serbia, Ilić et al. reported that iron-rich mica-containing kaolinite enhanced pozzolanic activity upon mechanical activation, whereby mica was identified as a contributing mineral in the kaolin.^8^

Geological deposits of kaolin are widespread across the world, with new exploration being commissioned.^9−13^ The study of new sites is important for evaluating end-use and to understand how the product can be improved relative to its mineralogy and mineral properties. Western Australia clay mineral deposits offer a wide range of kaolin minerals with various iron oxide content along with other impurities, including feldspar, mica, quartz, smectite, and illite. Recently, kaolin of varying quality was found in the Cloud Nine kaolinite–halloysite deposit, Noombenberry, Western Australia.^9^ However, understanding only mineral compositions of this clay are not sufficient to determine the economic potential of it. Large-scale deposits like Noombenberry, Western Australia showed diverse kaolin types, including tubular halloysite and platy kaolinite as well as their iron-bearing impurities.^14^ Knowing about these impurities and their possible effects on the range of clay modifications would be a benefit to understand more about this deposit and similar deposits elsewhere.

In this study, we sampled several kaolin clays based on their halloysite, kaolinite, and Fe contents. First, we applied state-of-the-art mineralogy, imaging, and spectroscopic techniques to understand the potential sources of iron impurities in kaolin samples. Second, considering the economic footprint of the clay processing, we did not apply any pretreatment such as purification, mineral separation, or fractionation, other than required to achieve the desired activation processes by acid activation and heating. Third, we assessed the morphology, surface charge, and gas sorption behavior to understand the effect of different mineral species and iron impurities on the samples’ physicochemical properties once they were activated.

## 2 Experimental Section

### 2.1 Materials and Chemicals

The kaolin samples were directly sourced from the Cloud Nine deposit, Western Australia, and supplied by the Latin Resources Ltd. (Table 1). The aim of this study was not to delineate between halloysite and kaolinite as both are polymorphs of the kaolin group. It is technically difficult to quantify them separately without a combination of tools, although machine learning technology has proven to be useful.^9^ However, based on Du Plessis et al.^9^ and an independent consulting report on the Cloud Nine deposit,^14^ a mineral composition specification was provided (Table 1). We received a database of clay mineral abundance for 100 drill hole profiles showing variations in mineral abundance. The key determinant was the percentage of halloysite, kaolinite, and iron oxides. Most of them categorically fall into six major groups, such as halloysite major, kaolinite major, or halloysite–kaolinite with a trace of iron oxides and counterparts having elevated amount of iron oxides (Table 1 and Figures S1 and S2). Other chemicals required for clay activation and pH adjustment were purchased from Sigma-Aldrich. These include reagent grade sodium hydroxide (NaOH) and hydrochloride acid (HCl). In this study, samples are referred to as LRS_H, LRS_HF, LRS_HK, LRS_HKF, LRS_K, and LRS_KF, where LRS refers to the mineral explorer, H stands for halloysite, K stands for kaolinite, and F stands for iron. Therefore, LRS_H would be halloysite rich clay, whereas LRS_HF is its counterpart with iron impurities. Other names follow the same system.

**Table 1:** Mineral Supplier-Provided Compositions of Selected Materials and Their Representative Categories

drill location^a^	depth (m)	code name	Hly (%)	Kln (%)	iron oxide (%)^b^	major kaolin species
NBAC358	28–30	LRS_H	58	20	0.32	Hly
NBAC375	22–23	LRS_HF	42	31	5.4	Hly
NBAC358	30–32	LRS_HK	39	43	0.32	Hly–Kln
NBAC353	32–34	LRS_HKF	28	39	5.71	Hly–Kln
NBAC369	9–11	LRS_K	20	76	0.38	Kln
NBAC378	29–31	LRS_KF	27	69	3.56	Kln

a The drill and sample points are provided as Figure S1.

b Types of iron oxides are unspecified as received and therefore considered as Fe_2_O_3_.

### 2.2 Modification of Raw Clay

The pristine clays as received were hand-crushed, passed through a 63 μm size sieve, and stored under dry vacuum conditions until further use. Three types of modification of the six materials were performed, namely, (i) acid treatment, (ii) heat treatment, and (iii) heat treatment of the acid-treated samples.

#### 2.2.1 Acid Treatment

The clay was treated with HCl (3 M) following a revised method reported elsewhere.^15,16^ In brief, 1:10 (w/v) of clay to acid solution was made and agitated (∼100 strokes/min) in a 70 °C hot water bath for 2 h. The dry clay powder was regained after repeated centrifugation, washing with fresh Milli-Q (MQ) water, and 60 °C hot oven drying for 48 h. While the supernatant was kept for measuring dissolved iron, the treated material was stored in a desiccator until completion of full characterization.

#### 2.2.2 Heat Treatment

Using a muffle furnace, the clay was heated at 600 °C for 4 h with no N_2_ or air flow.^17^ The material remained inside until it cooled down to ∼100 °C, followed by its further cooling in the desiccating chamber. Characterization was performed intermittently using the stored samples.

As a third modification, we also performed the same thermal treatment of the acid-activated product described in Section 2.2.1.

### 2.3 Characterization of Raw and Modified Clays

#### 2.3.1 Mineral Assemblage Recognition

Random powders of raw and modified clays were prepared by packing into a sample holder. These were analyzed with an X-ray diffractometer (XRD) (PANAlytical Empyrean, The Netherlands) with a Cu X-ray source. We did not apply any pretreatment for this sample preparation. The raw pattern was used to identify mineral content using the X’pert HighScore software and the database PDF-4/Minerals 2022, ICDD.^18^ We also performed Fourier transform infrared spectroscopy (FTIR) of potassium bromide-mixed pellet samples to better understand the mineral and impurities associated with the studied clay samples. For this, we used a PerkinElmer FTIR spectroscope in the 4000–400 cm^–1^ scan range.

#### 2.3.2 Bulk Sample Composition of Raw and Modified Clays

Using a PANalytical Eplison 1, X-ray fluorescence spectroscopy (XRF) analysis of the powdered materials was performed. Only oxides were selected, which were normalized to 100% without the loss of ignition values included.

#### 2.3.3 Iron Content, Iron Oxide States, Acid Dissolution, and the ^27^Al Solid-State NMR

To locate the position of iron in the samples, we mapped microscopic areas dominated by halloysite or kaolinite for the element of interest using energy-dispersive spectroscopy (EDS) (Bruker EDS system, U.K.). The total dissolved Fe and other selective elements (Al, Si, Ca, and S) from the first two washes of the acid treatment process (Section 2.2.1) were measured using inductively coupled plasma optical emission spectroscopy (ICP-OES) (Avio 200, PerkinElmer Instruments). X-ray photoelectron spectroscopy (XPS) (Kratos AXIS Ultra DLD, U.K.) was also used to detect the state of iron oxides in the samples. To reveal the chemical shift of aluminum (Al) from contrasting Fe-rich clays after the acid treatment, a solid-state nuclear magnetic resonance (NMR) measurement was carried out using a Bruker Avance III 300 mHz instrument operating at a frequency of 78 mHz for the ^27^Al nucleus. The samples were packed in a 4 mm zirconia rotor and spun to 12 kHz at the magic angle. The spectra were gained with a hard 3 μs pulse and with 1k signal transients for a sufficient signal-to-noise ratio. The spectra were referenced to the ^27^Al signal of a 1 molar aqueous solution of Al(NO_3_)_3_ at 0 ppm.

### 2.4 Physicochemical Properties and Outcomes

#### 2.4.1 Microscopic Images of Clays

Platinum-coated samples, 4 nm in thickness, were imaged by scanning electronic microscopy (SEM) at various magnifications (Zeiss VP Sigma, Germany). To image the changes in particle shape, selective samples were studied by transmission electron microscopy (TEM) using a JEOL-2100F, Japan. Using imageJ software (version 1.53K), ∼10 random particles were taken for measuring the length of the halloysite tube and diameter of the tube and lumen.

#### 2.4.2 Surface Charge (ζ-Potential) of Raw and Modified Clays

The ζ-potential employed as a proxy of the particle surface charge was measured using a NanoPlus-HD under natural/controlled pH values. For this purpose, a diluted colloidal suspension (0.05% in MQ water) was used.

#### 2.4.3 Pore Size, Surface Area, and Gas Sorption

Surface areas, pore volume properties, and the N_2_ gas sorption isotherms of degassed samples were measured using a surface area and pore analyzer (Micromeritics Tristar II-3020). The specific surface area (SSA) and the maximum gas sorption capacity were reported based on the Brunauer, Emmett, and Teller (BET) theory, whereas the pore size distribution was determined by the Barrett–Joyner–Halenda (BJH) model for the adsorption phase of the gas.

## 3 Results and Discussion

### 3.1 Key Properties of Samples Relating to Iron Impurities

#### 3.1.1 Mineral Assemblages and Composition by XRD and XRF

**Figure 1:**
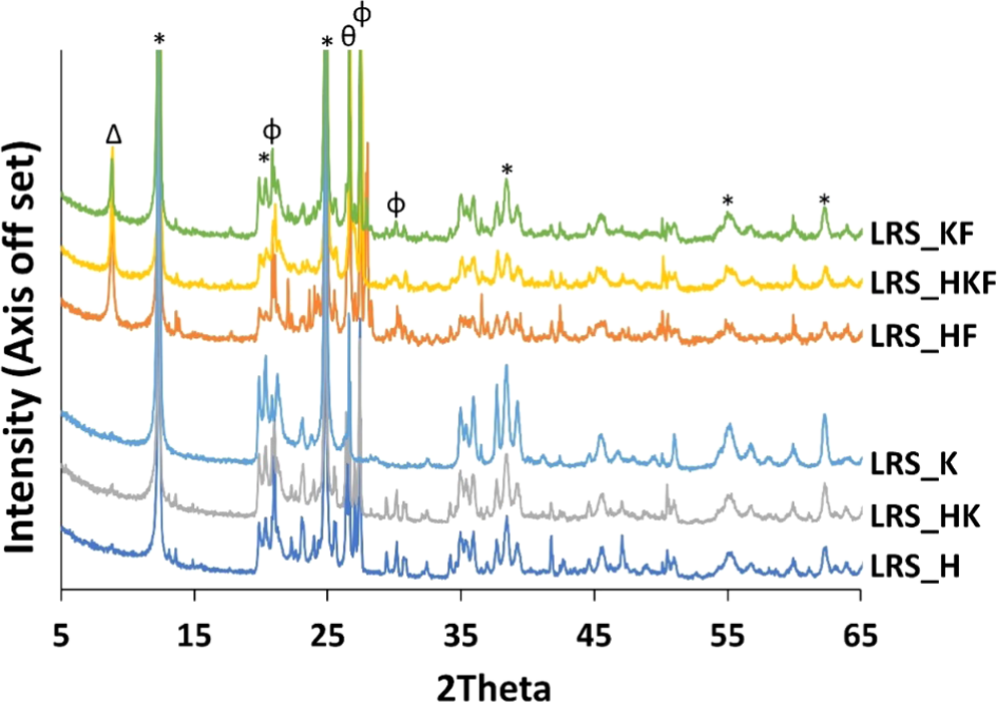
Full pattern XRD of raw samples. * = kaolin; Φ = K-feldspar; θ = quartz; Δ = mica/illite (iron-bearing non-kaolin mineral). FTIR spectra are provided in Figure S3.

All six samples were dominated by kaolin group minerals. These minerals are characterized by a XRD *d*_001_ reflection at 7.1 Å (2θ = 12.4°). Feldspar and quartz were detected as impurities (Figure 1). Other impurities like carbonates were not detectable in the studied samples (Figures 1 and S3). The presence of oxides of aluminum and silicon was confirmed by XRF as the dominant oxides in the octahedral and tetrahedral layers of the clay minerals. When iron was detected in the samples (Tables 1 and 2), mica or illite (*d*_001_ = 10.0 Å at 2θ = 8.8°) appeared to be associated with the kaolin minerals (Figure 1).

**Table 2:** XRF Profile of the “Major Oxides” of Raw Samples

	LRS_H	LRS_HF	LRS_HK	LRS_HKF	LRS_K	LRS_KF
Al_2_O_3_	36.19	29.80	38.55	30.08	40.53	36.90
SiO_2_	55.73	56.48	54.71	56.78	56.78	54.41
Fe_2_O_3_	0.56	6.07	0.40	5.33	0.57	3.74
TiO_2_	0.46	0.47	0.66	2.00	0.67	0.32
K_2_O	5.96	4.68	4.72	4.30	0.38	3.59
P_2_O_5_	0.517	0.66	0.505	0.713	0.528	0.511

Similar mica associations were also reported in XRD patterns for a Serbian kaolin deposit, which also contained ca. 3.78% of iron oxides.^8^ Studying Batn El-Ghoul kaolin collected from South Jordan, Gougazeh reported a similar source of iron.^19^ In our case, we also detected ∼3.74% of iron oxide (XRF predicted value) in kaolinite rich samples, and even greater amounts in the halloysite dominated (Table 2). In the AlO_6_ region, the content of Al_2_O_3_ negatively correlates with the abundance of Fe_2_O_3_ because of the isomorphic substitution in the 2:1 clay minerals like illite.^20^ This is supported by the XRF profile of Al_2_O_3_ (Table 2) where the iron-rich variant of halloysite or kaolinite has a lower amount of Al_2_O_3_ than its iron-poor counterparts.

#### 3.1.2 Imaging Minerals and Iron and Its States by SEM-EDS and XPS

**Figure 2:**
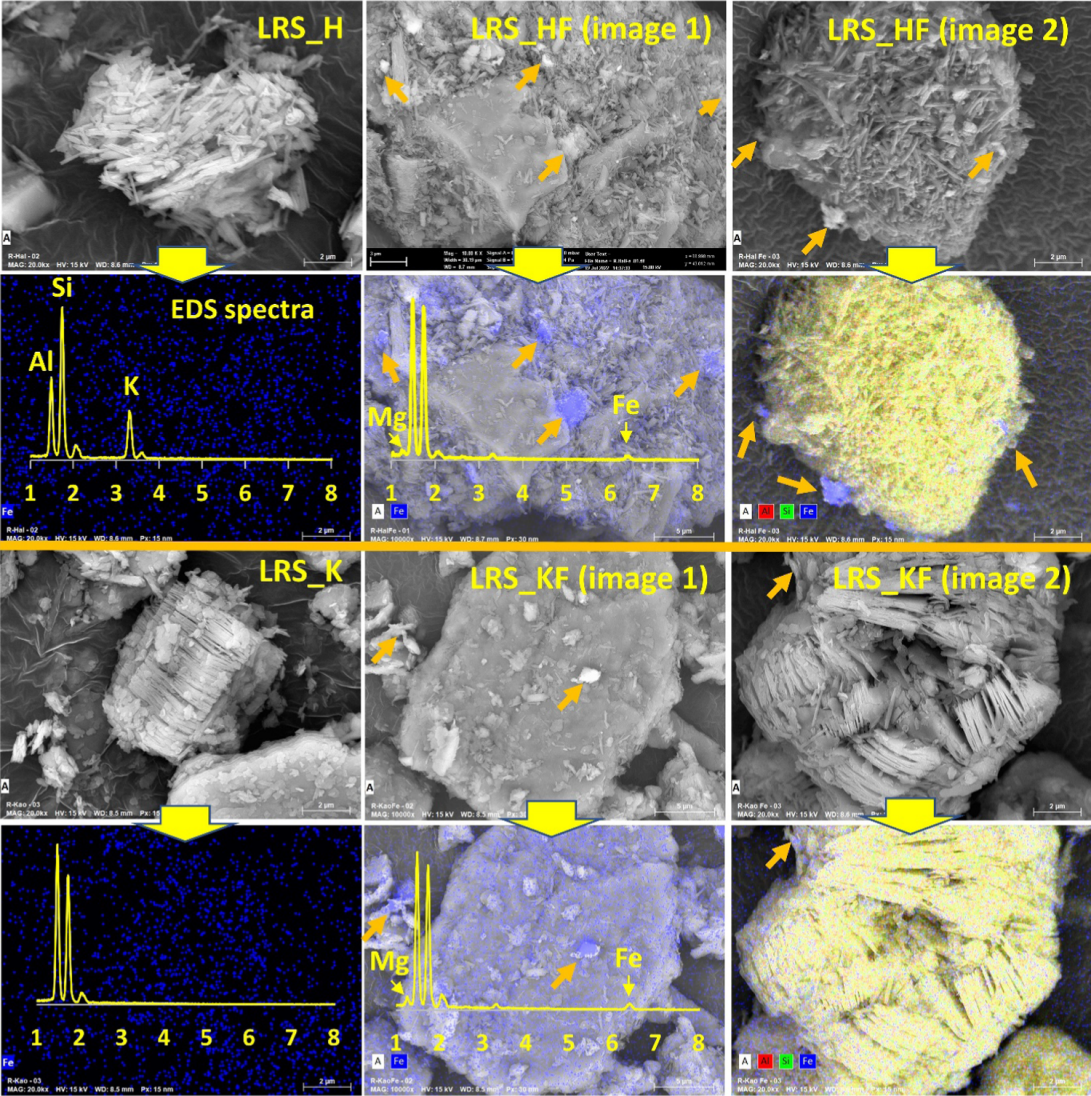
Imaging mapping of whereabouts of iron in the iron-rich minerals. The orange arrow shows the tracing of iron in the materials, which is additionally confirmed by the EDS spectra.

The EDS maps showed the existence of iron at a submicron level with the particle arrangements (Figure 2). In the pristine LRS_H or LRS_K, only the background noise of the iron signal was detected, showing an insignificant scattering of iron. Conversely, in the LRS_HF and LRS_KF, the iron was present and significantly localized—a sign that the source of iron was from selective mineral species (Figure 2).

**Figure 3:**
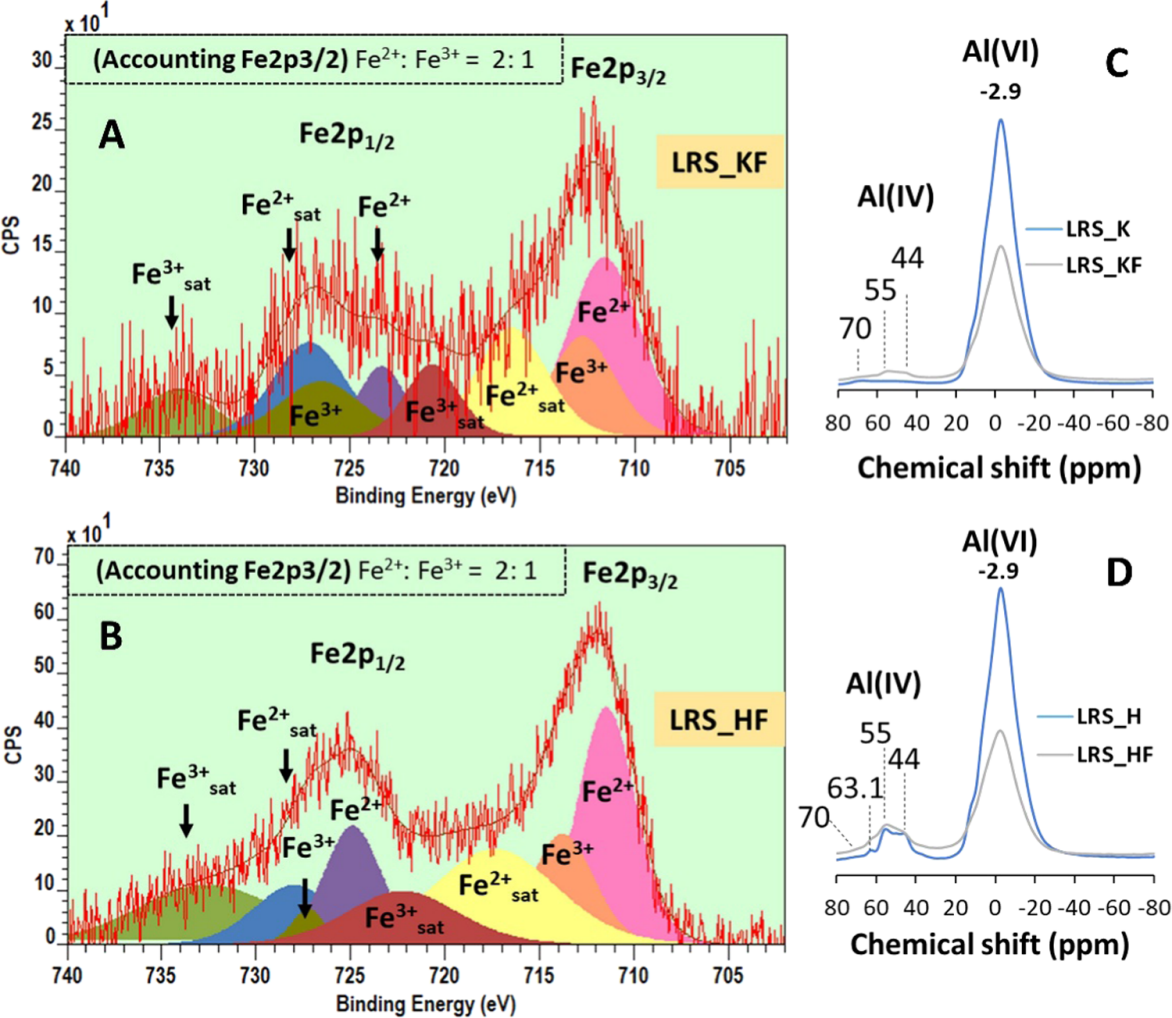
Component model of XPS spectra for the Fe 2P regions and ^27^Al NMR spectra of samples. For XPS, (A) LRS_KF and (B) LRS_HF. Other samples, which are iron-deprived did not produce high-resolution Fe 2p peaks, and therefore, no modeling was performed. For NMR, (C) and (D) represent LRS_K and LRS_H and their iron-rich counterparts, respectively.

To understand the oxide states of iron, we performed XPS on samples and detected that the iron oxide linked to the mica/illite is predominately Fe^2+^ and approximately twice the Fe^3+^. There was no elemental Fe detected (Figure 3). Stucki et al.^21^ studied biotite, a trioctahedral mica and suggested Fe^2+^ to be predominant over Fe^3+^. Further, the NMR spectra of the studied halloysite and kaolinite and their iron-rich varieties showed the existence of a non-kaolin mineral species. The raw kaolin clay contained mainly Al octahedral (six-coordinated),^22^ and in our case, that is at −2.9 ppm (Figure 3). Usually, kaolin (kaolinite and halloysite) exhibited a poor intensity (<1%) in the Al(IV) region.^23^ When iron as a major coexisting oxide is present, the four-coordinated Al became apparent along with the six-coordinated (AlO_6_) one. Therefore, significant resonance in this region might be linked to the impurities such as mica and illite. For example, muscovite resonates at 70 ppm, feldspar at 63.1 ppm, and/or illite at 44–55 ppm. Here, compared to LRS_K, LRS_H has higher amount of these associated minerals. This is further supported by the XRD reflection intensity of the *d*_001_ spacing located at 2θ = 8.8° where the value is greater for LRS_H than for LRS_KF (Figure 1 and Table S1).

### 3.2 Material Properties of Samples after Activation

#### 3.2.1 Mineral Assemblages

**Figure 4:**
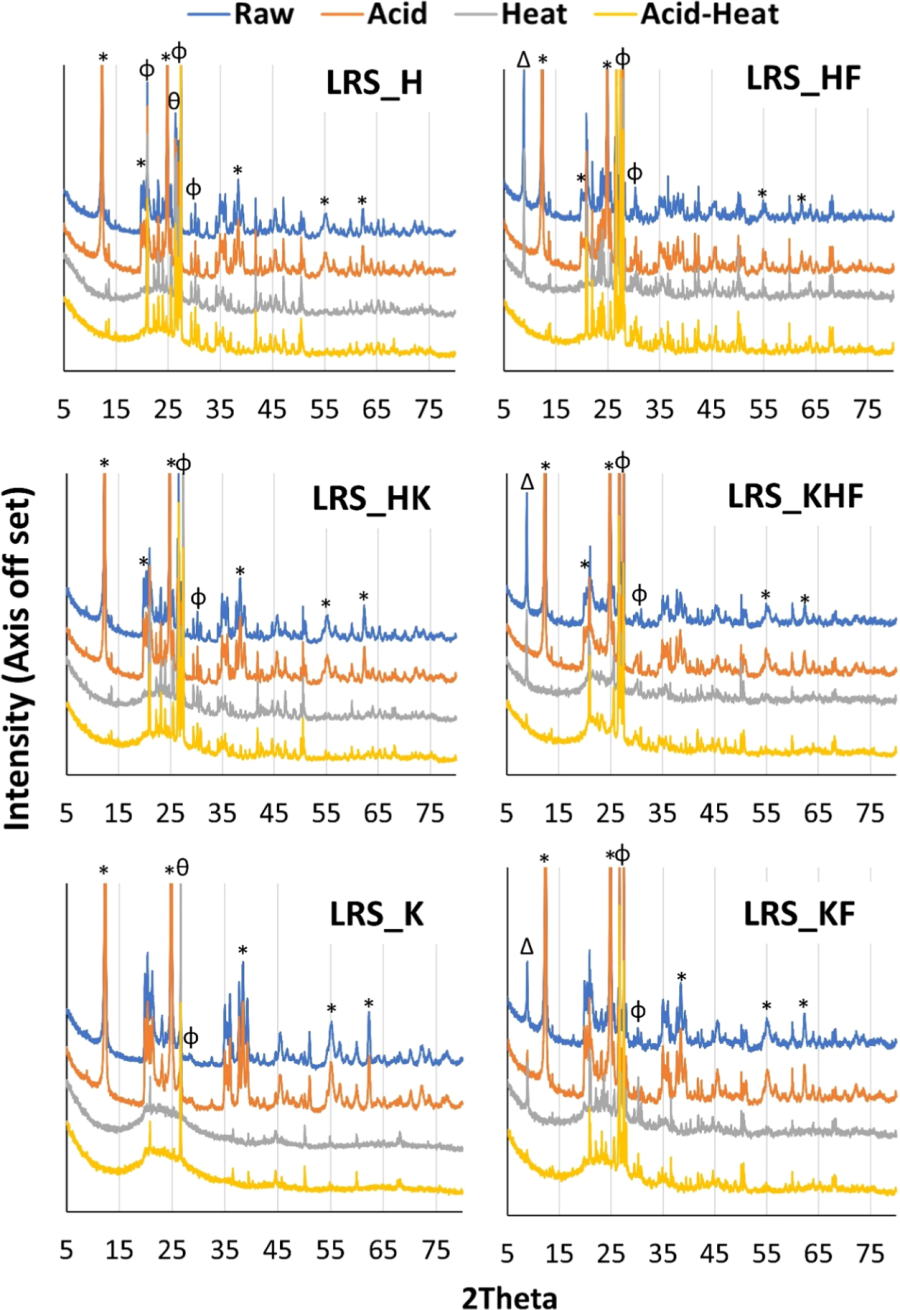
Full pattern XRD of raw and modified kaolin materials. * = kaolin; Φ = K-feldspar; θ = quartz; Δ = mica/illite (iron-bearing non-kaolin mineral).

With various physicochemical activation processes, the acid treatment did not affect the primary reflection of kaolin minerals. However, the heat activation caused a significant change (Figure 4 and Table S1). The thermal modifications of all clay samples at 600 °C led to the absence of kaolin *d*_001_ reflections, regardless of them being major halloysite, kaolinite, or with their iron oxide impurities (mica/illite). The dehydroxylation of kaolin mineral occurred in this case, reportedly between 400 and 700 °C depending on thermal and sample conditions.^24^ This alternation causes the breakdown of silica and alumina polyhedra in their tetrahedral and octahedral sheets, and results in the amorphous state of the kaolin minerals.^25,26^ In contrast, micas do not become fully amorphous after dehydroxylation at temperature ca. >800 °C but remain in a structurally modified state.^27,28^

With acid treatment, the activation conditions are expected to influence the mineral characteristics observed. These include types of acid used, temperature or physical force like stirring. For example, Zhou et al. used sulfuric acid to activate kaolinite (geological source: Longyan, China) and coal-bearing kaolinite (geological source: Datong, China) and reported a minor reduction of peak intensity at *d*_001_ and an enlargement in the interlayer space.^29^ Here, we recorded no or insignificant changes in peak intensities or the *d*_001_ spacings (Table S1). Taking 1 g of clay into 10 mL of 3 M HCl, we shoke the mixture only at 50 strokes/min for 2 h at 70 °C that are quite comparable to that applied by Zhou et al.^29^ Also, acid attack on mica/illite was significant and effective, resulting in the weakening of the *d*_001_ XRD reflection of these minerals (Figure 4). It shows that the protonated medium affected the structural integrity of mica/illite and accelerated the dissolution of metal ions, including the associated Fe(II) and Fe(III).^30^

#### 3.2.2 Changes in the Relative Amount of Major Oxides

The dissolution of iron is a common effect of the hydrothermal modification of clay minerals. In our cases, the acid treatment dissolved it more than by the heat treatment alone (Table 3), which was further enhanced by an additional calcination temperature. For example, XRF study of raw and modified clays detected that the loss of Fe_2_O_3_ in heat-activated LRS_H was 7.31% that is marginally lower than that for acid activation. However, while LRS_HF was assessed, this difference was significant (Table 3). This trend was identical to other pairs of clay variants, such as LRS_K vs LRS_KF or LRS_HK vs LRS_HKF.

**Table 3:** Iron Oxide of Raw and Modified Kaolin Minerals^a^

		S_i_O_2_/Al_2_O_3_	Fe_2_O_3_ (% of all oxide)	Fe_2_O_3_ decrease^b^	acid-leached Fe (mg/g)^c^
LRS_H	raw	1.54	0.56		
	heat	1.40	0.52	7.31	
	acid	1.51	0.51	8.73	0.59 ± 0.02
	acid-heat	1.44	0.45	19.79	
LRS_HF	raw	1.90	6.07		
	heat	1.69	5.22	14.09	
	acid	1.97	1.48	75.67	24.42 ± 4.05
	acid-heat	1.94	1.34	77.89	
LRS_HK	raw	1.42	0.4		
	heat	1.34	0.34	14	
	acid	1.40	0.3	26	0.78 ± 0.005
	acid-heat	1.35	0.26	35.75	
LRS_HKF	raw	1.89	5.33		
	heat	1.79	4.84	9.24	
	acid	2.00	1.61	69.91	28.68 ± 3.26
	acid-heat	1.94	1.5	71.88	
LRS_K	raw	1.40	0.57		
	heat	1.15	0.45	20.42	
	acid	1.23	0.46	19.01	0.31 ± 0.02
	acid-heat	1.17	0.42	26.58	
LRS_KF	raw	1.47	3.74		
	heat	1.33	3.24	13.47	
	acid	1.49	1.34	64.12	14.22 ± 1.23
	acid-heat	1.38	1.32	64.81	

a Full XRF tabulating possible metal oxides is presented as Table S2.

b Relative to the raw material.

c Quantified directly by ICP-OES.

#### 3.2.3 NMR Spectroscopy for Al and Acid Dissolution of Cations

**Figure 5:**
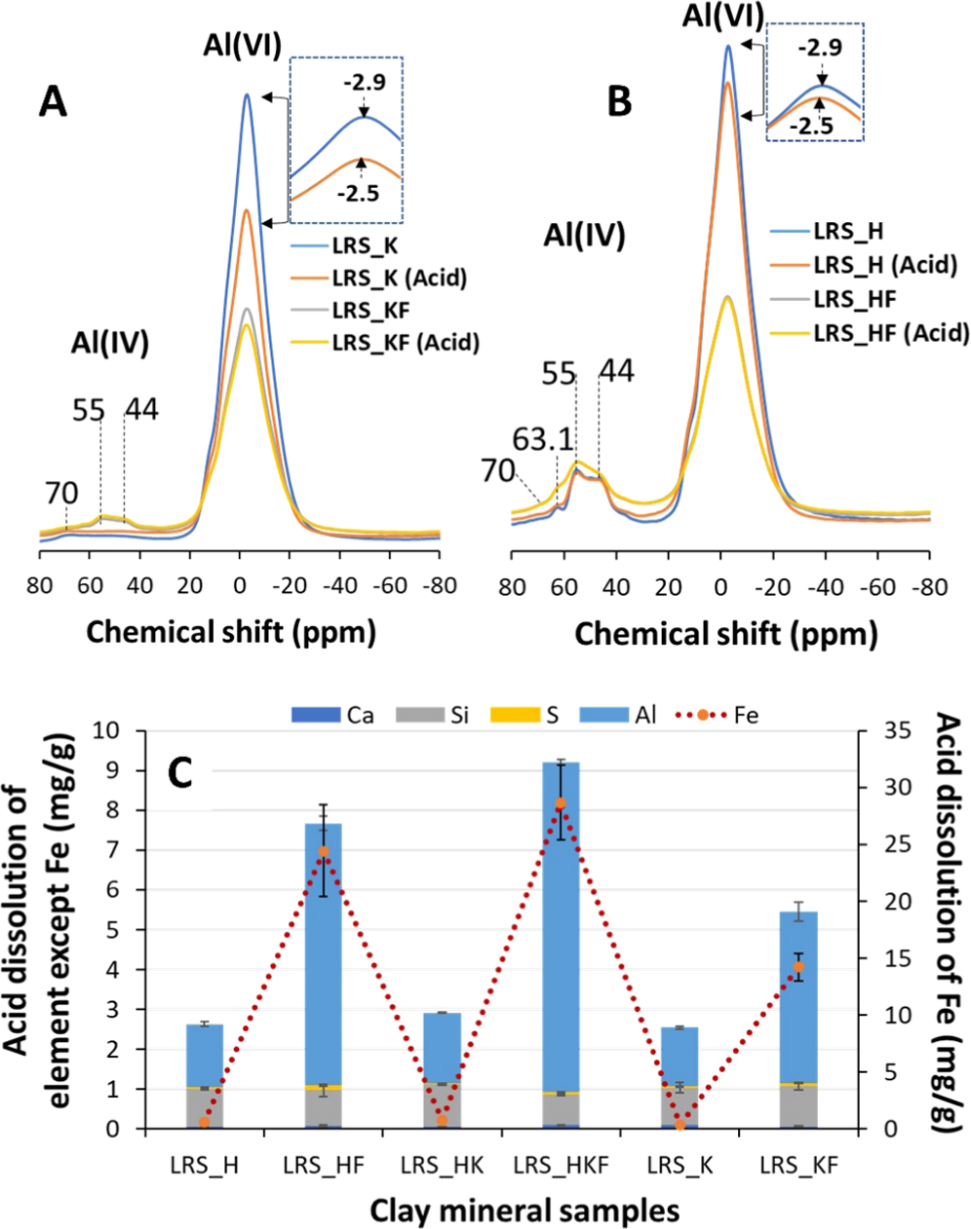
^27^Al NMR of the raw and acid-treated (A) LRS_H and (B) LRS_K samples. (C) Absolute concentration of dissolved cations in the acid-treated samples.

Considering that the acid activation has the most effect on the dissolution of Al and Fe, we only performed NMR for selective raw and acid-treated samples. A little or apparently no chemical shift (at −2.5 ppm from −2.9 ppm) occurred after acid treatment (Figure 5).^22^

Acid treatment of the studied clays lowered the intensity of the AlO_6_ region, while the AlO_4_ remained conserved. In a 2:1 clay like illite, tetrahedral Al is stable against acid attack as it is positioned in the three-dimensional framework of the siloxane sheet. In contrast, octahedral Al that is exposed at the edge of the crystal structure encounters the H^+^ in the acid medium.^31^ The Al concentration (total) further supports this dissolution potential (Figure 5).

### 3.3 Mechanistic Insights into Clay Activation Linked to Iron Impurities

Iron is present in the mica/illite of the kaolin samples collected from the Cloud Nine deposit, WA. While materials heated to ca. ∼600 °C, kaolin minerals modified to metakaolin, and acid activation leached the iron-bearing mica/illite. The oxidative Fe is susceptible to be dissolved compared to its other forms.^30^ They reported that the dissolution rates of iron were attributed to mineral types, claiming the order of pyrite > illite > chlorite > kaolinite.^30^ It is worth mentioning that we identified iron in the “kaolin” samples nested in the lattice of non-kaolin minerals, in particular in that of, mica/illite (Figure 1). This supports the possibility that the variety of minerals is the driving factor for the iron dissolution.^30^ Indeed, if we compare pairwise the samples such as LRS_H vs LRS_HF (Table 1 and Figure 1), the amount of iron and the non-kaolin mineral generally correlate.

Considering the NMR pattern of Al in the octahedral region (Figure 5) and the positive relation between the leaching of Al and iron (Figure 5), one would expect that the dissolution of Fe is linked to the octahedral region of mica/illite.^32,33^ Therefore, the hydroxyl-bearing sheets of the mica/illite might be the major site of iron dissolution. An additional link between the oxides of potassium and iron could provide further clues to the location of iron impurities. For instance, once the acid modification was completed, the percentage loss of K_2_O in the iron-rich samples followed the same trend as Fe_2_O_3_ (Tables 3 and S2). While mica (e.g., muscovite) and illite have potassium as the main interlayer cation, loss of both K and Fe together indicate a significant dissolution of mica/illite in these samples.^19^

### 3.4 Physicochemical Property Outcomes from Activated Clays

#### 3.4.1 Morphology of Modified Clay Minerals

**Figure 6:**
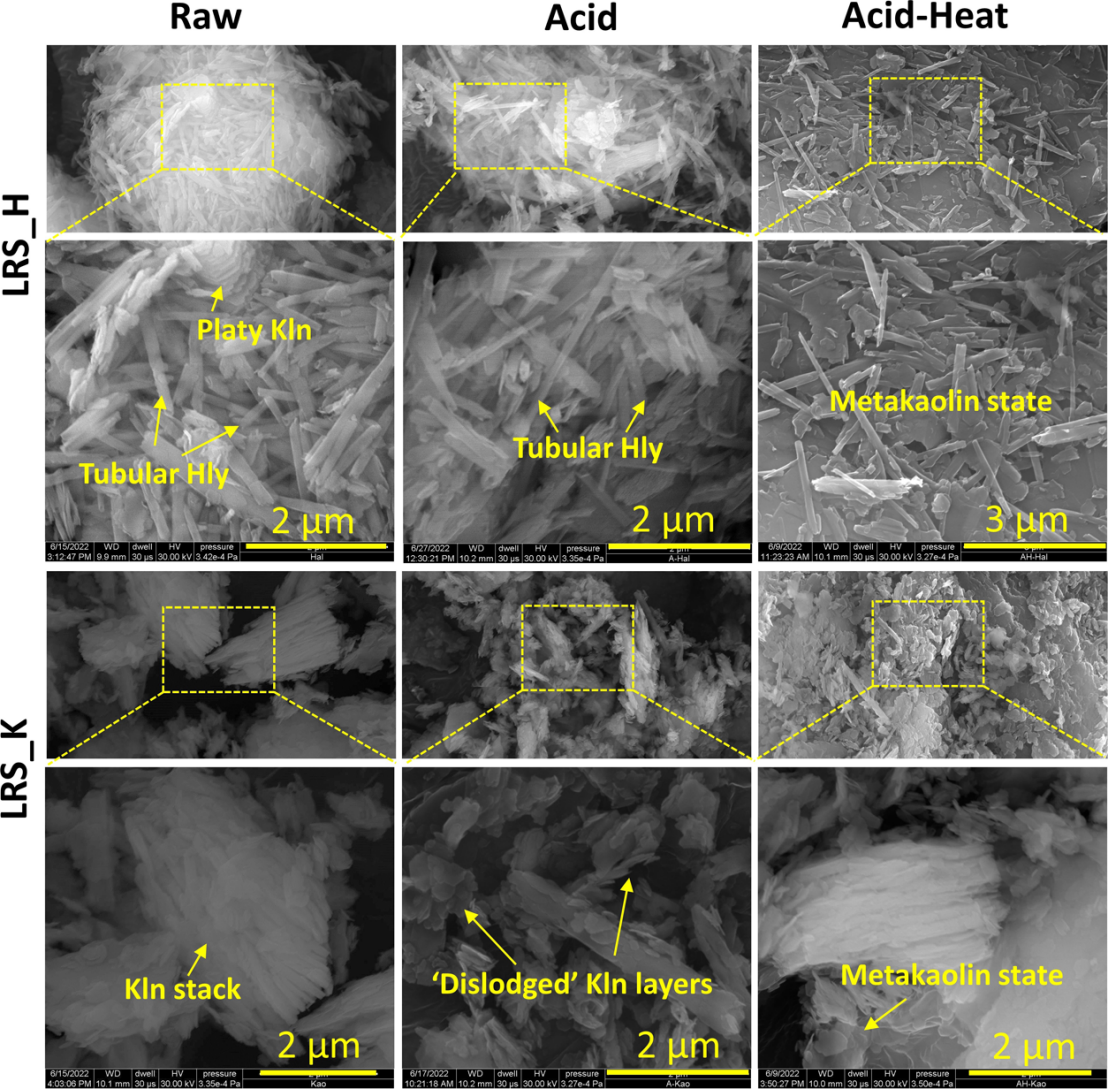
SEM images of the pristine and modified clays. Only type “LRS_H” and “LRS_K” have been presented in this figure as a comparison among the physicochemical treatments. The box in the low-magnification image does not indicate the exact point and area of imaging of the high-magnification one. Images of other variants are provided in Figure S5.

Halloysite was identified to be nanotubular, whereas the kaolinite had a typical sheet and stack arrangement in the samples. A relatively small crystallite size of the kaolinite-rich species (∼24 nm at in the crystallographic *c*-direction) suggests a poorly ordered structure, while comparatively a high value of this parameter (∼37 nm) probably reflects the large tubular sizes of halloysite (Table S1).^34^ The halloysite was 1035 ± 606 nm long, while the tube and lumen diameters were 153.6 ± 41.9 and 40.7 ± 12.9 nm, respectively (Figure S4). In contrast, the most widely used commercially available Sigma-Aldrich halloysite has a much narrower lumen diameter (∼13 nm).^35^ We achieved a 52 ± 17.0 nm width of lumen after the acid treatment of the nanotubes (Figure S4). However, the acid activation did not disrupt the structure of halloysite or kaolinite (Figure 6). Rather, it tended to dislodge the aggregates of halloysite nanotubes and the stacks of kaolinite. Similar to a prior study, we also observed an increasing number of kaolinite flakes that were peeled off as layers in the acid-treated samples.^30^ In contrast, temperature treatment caused significant changes in the clay minerals, as also seen in XRD patterns (Figure 4). It is mainly due to dehydroxylation that produces amorphous metakaolin of the kaolin clay (Figure 6).

#### 3.4.2 Surface Area and Charge of Activated Samples

The acid treatment increased the SSA to varying degrees depending on the kaolin type and Fe impurities. That is in contrast to the heat activation of clay materials (Table 4).

**Table 4:** Specific Surface Area (SSA) and ζ-Potential Value

	raw		acid		heat		acid-heat	
samples	SSA (m^2^/g)^a^	ζ (mV^)^^b^	SSA	ζ	SSA	ζ	SSA	ζ
LRS_H	9.86	–23.58	10.76	–41.66	8.38	–33.91	10.19	–31.02
LRS_HF	9.94	–18.6	20.45	–39.88	8.79	–42.38	18.80	–50.98
LRS_HK	11.56		10.89		9.49		11.34	
LRS_HKF	25.11		32.55		20.75		32.00	
LRS_K	14.17	–44.91	13.85	–39.08	12.75	–46.12	13.95	–35.69
LRS_KF	11.12	–36.11	13.98	–47	9.25	–43.5	12.71	–40.24

a At ∼0.291 *P*/*P*_0_ single point based on the BET theory.

b Results obtained for the key mineral samples at pH of ∼6.0.

Overall, the kaolinite as a major component of the samples did not show a significant increase in SSA compared to the halloysite-rich samples. It is reported that the etching effect caused by the availability of H^+^ dissolves Al from the octahedral layer, resulting in an increasing internal surface area.^36^ Considering this, one would expect that the acid-treated LRS_H sample should provide quite an increased SSA (Table 4). The SSA of this acid-treated sample was only 10 m^2^/g from the original value of ca. 9 m^2^/g in LRS_H. In contrast, the same treatment caused a significant change LRS_HF (∼20 m^2^/g from the original value of ∼9 m^2^/g in LRS_HF vs ∼). This iron-rich halloysite sample has over 5% Fe_2_O_3_—that is quite significant—and its removal may have led to a proportional concentration increase of pure halloysite with a high SSA for this material (Table 3). Inherently, low kaolin-rich clay with iron-rich particles (LRS_HKF) produced the greatest SSA value (∼25 m^2^/g) among all clay samples studied because of proportionally large contribution from iron oxide and iron-bearing minerals like mica/illite. In this case, the leaching of Fe increased SSA only to a moderate degree but not to the extent observed in LRS_HF. This supports the idea that the relative etching of the octahedral layer of halloysite had a significant role in generating additional SSA in the halloysite-rich samples studied.

We also determined the ζ-potential values of key samples at pH ∼6.0 for the material comparison (Table 4). The pH of MQ water solution of studied raw clays was ∼6.5. Unlike the SSA, the ζ-potential values of kaolinite-rich samples were higher than their halloysite counterparts (e.g., −44.91 for LRS_K vs −23.58 for LRS_H). Once they were modified, types of activations and mineral impurities, like iron-bearing mica/illite, contributed to the net surface changes. As a result, all modified clays showed some degree of increased negativity in their surface charges (Table 4).

#### 3.4.3 Pore Volume Properties

**Figure 7:**
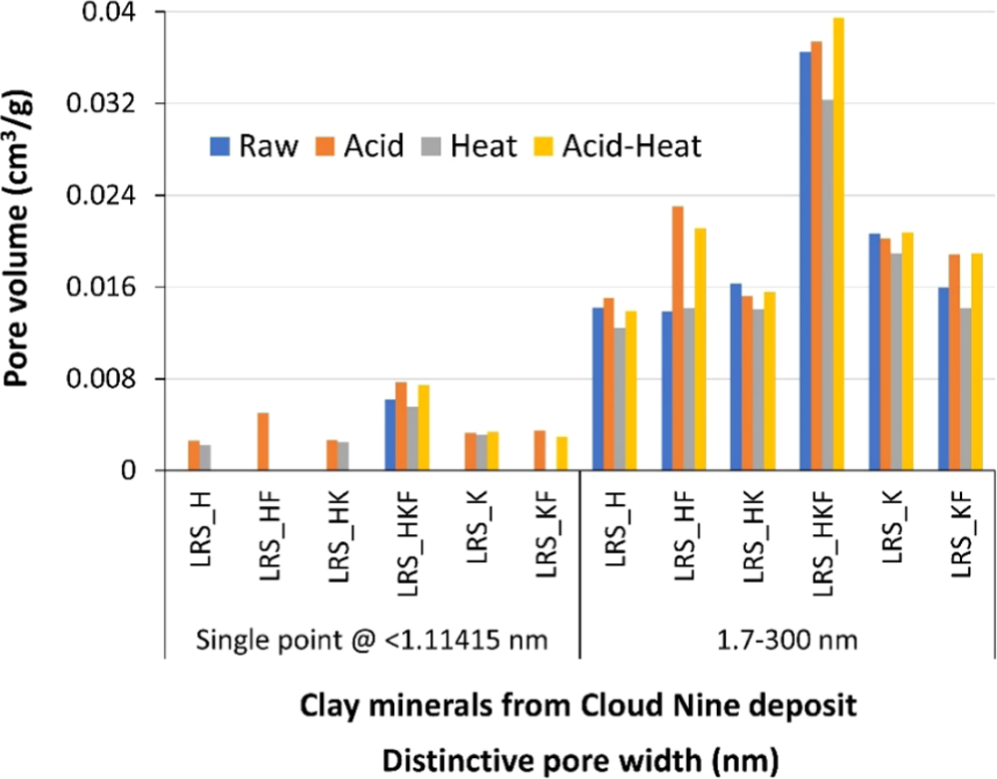
Pore volume relative to the micro- and mesopore regions affected by the various physicochemical treatments of the raw clay mineral samples. The pore width categories are the theoretical values suggested by the surface area analyzer report (TriStar II-3020 Version 3.02).

Pore volume profiles matched well the pattern of SSA that occurred in response to various activation processes (Figure 7). We presented two distinct pore volume profiles: (i) cumulative adsorption by the BJH theory from 1.7 to 300 nm pore diameter and (ii) across the micropore region considering a single point adsorption. Except for LRS_HKF, pores were generated in the mesopore size-range. The greatest increase in the pore volume was 0.0230 cm^3^/g for acid-treated LRS_HF compared to its raw counterpart (0.0138 cm^3^/g). The space formed in the micropore region of the materials containing mica/illite and even more in samples where halloysite was the dominant mineral (Figure 7). This is in accordance with the SSA changes observed in the same material and its modification (Table 4).

### 3.5 Discussion Relating New Properties to the Activatation of Clays

Acid treatment is proven as an effective method for enlarging the lumen diameter of the halloysite nanotube.^37,38^ However, random particle measurements indicate that iron impurities did not affect these changes in the lumen, indicating that the presence of mica/illite did not prevent this process. However, these non-kaolin minerals may have prevented the bulk of the clay becoming amorphous by conversation of kaolin to the metakaolin state that occurs during moderate heating (∼600 °C).

In terms of attaining desirable surface charges of the bulk particles, removing or retaining of key cations is a key consideration. In this context, the iron oxide content may increase the surface positivity of halloysite and kaolinite.^39^ However, the proportion of SiO_2_ and Al_2_O_3_ in the metakaolin samples might be another factor. Siloxane groups contribute to elevate the negative charges in water while bonding to surface hydroxyl groups.^40^ Acid treatment removed cations, especially Fe from the mica/illite-mixed samples and increased the negative ζ values of the samples (Table 3). However, the pH dependence of surface charges matters (Figure S6). In this case, the basal surface charges play a role; faces that carry negative charges tend to be permanent, while those at the edges are pH variable.^41,42^ The studied samples exhibited a negative charge in a water over the range of pHs where the kaolinite-rich variants of clays were probably inherently exposed with more net negative surfaces in the aqueous suspension.^40^

**Figure 8:**
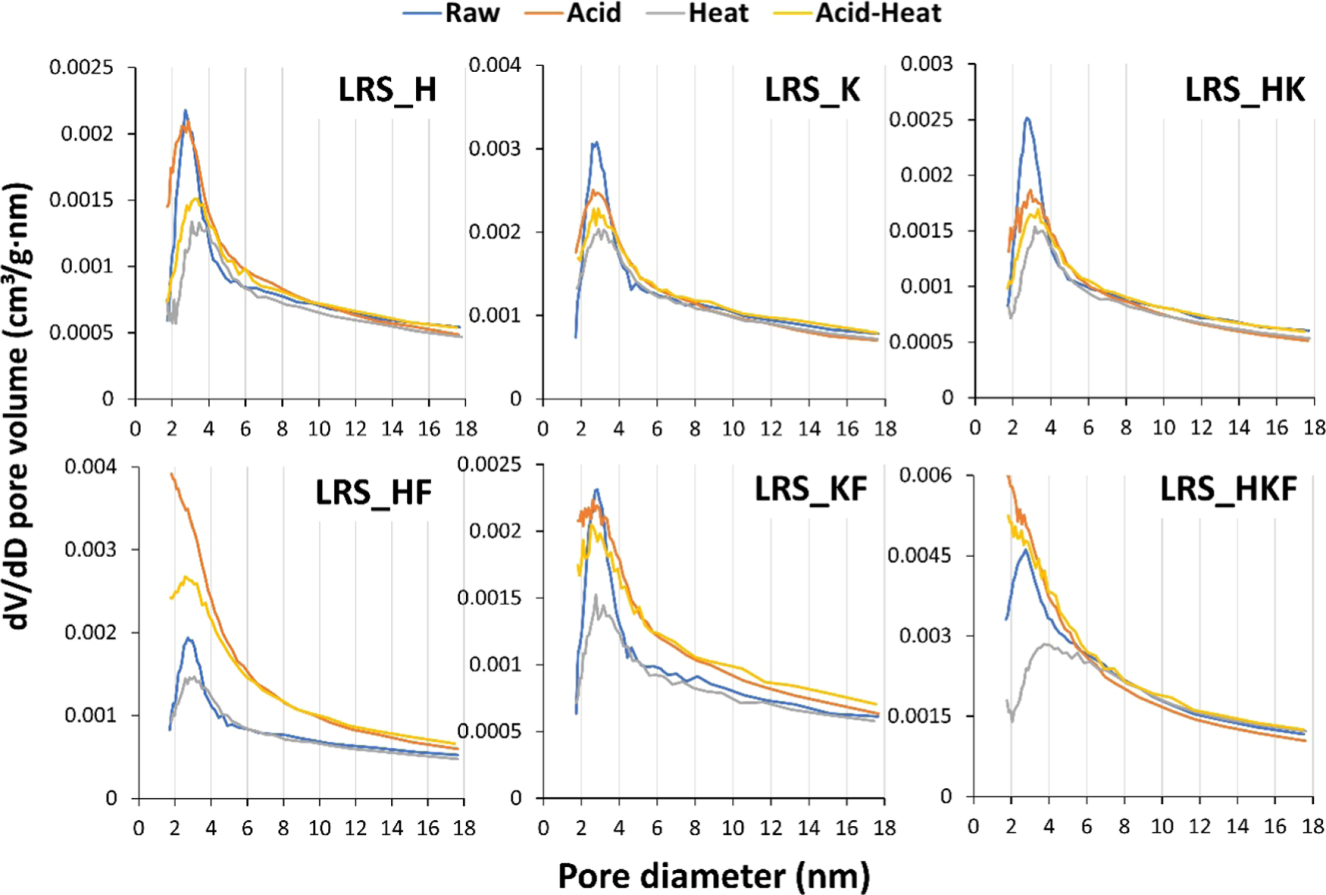
Differential pore volume against the pore diameter profile of the raw and activated clay minerals.

The major increase in SSA and pore space occurred after the iron impurities were removed by the acid treatment, which was particularly the case for halloysite-dominated samples. The increase in new pore volume resulted mainly in the range of narrow mesopores. Figure 8 shows that the pore diameter of 2–3 nm produced the most void space once the LRS_HF was activated with 3 M HCl but this was subsequently reduced by the heating. In contrast, although acid treatment enhanced the pore volumes between LRS_KF particles, this sample behaved differently in terms of the size of the resulting pores. Pores over 3 nm diameter contributed significantly to forming void spaces in LRS_KF (Figure 8).

Two distinct features play a role in the distribution of pores that characterize the kaolin minerals. They are (i) the length-to-size aspect and the etching of lumen, for halloysite, and (ii) acid resistivity of a platy structure, for kaolinite.^43^ Regardless of the types of kaolin, the desirable outcome for SSA and pore modification relies on the “processing” conditions applied to the starting materials. For example, Pasbakhsh et al.^44^ studied halloysite from South Australia, Western Australia, and New Zealand and recorded an increase of more than double the SSA and pore volumes than that we report for the Cloud Nine kaolinite–halloysite deposit of Western Australia. They applied mild alkali treatment and a sedimentation process to obtain <2 μm clay particles. In contrast, we did not apply any processing or pretreatment, and thus the achieved properties are not directly comparable due to the processing differences of the starting material. It is worth noting that the removal of carbonate impurities by acid treatment could also increase SSA and pore sizes of clay;^45^ however, the absence of measurable carbonates in the studied kaolin suggests that this effect is here negligible (Figure S3).

### 3.6 Gas (N_2_) Sorption and Mechanistic Insights Linked to Modified Properties

**Figure 9:**
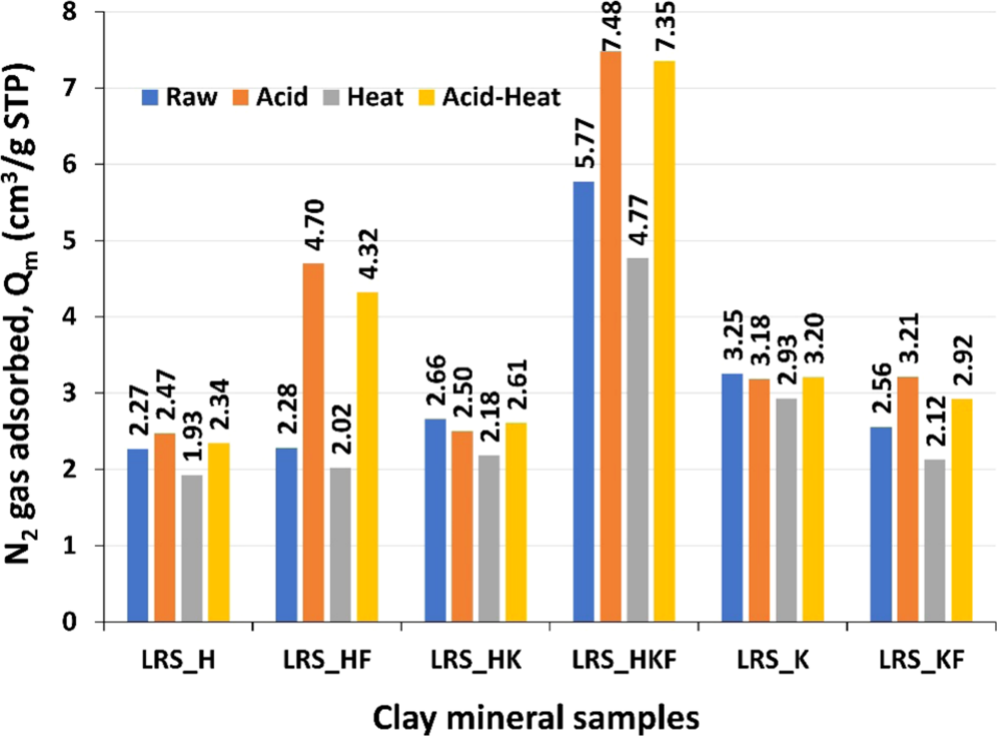
Maximum gas adsorption capability in the form of monolayers calculated based on the BET at the standard temperature and pressure (STP).

The adsorption capacity of the studied materials is linked to the SSA and pore volume (Figure 9). Here, only the thermally treated clays did not provide enhanced properties for improved gas molecule attraction. In contrast, acid dissolution of clays, in particular of those that are iron-rich, adsorbed a greater amount of gas molecules. For example, acid activation produced a *Q*_m_ value of 4.70 cm^3^/g compared to its pristine counterpart LRS_HF (2.28 cm^3^/g). Conversely, it was only 2.47 cm^3^/g (acid-activated LRS_H) against its pristine LRS_H (2.27 cm^3^/g).

We also measured isotherm curves and hysteresis loops (Figure S7). In this study, the hysteresis curves are identified as H3. According to the International Union of Pure and Applied Chemistry (IUPAC), this H3 loop is created by the type II adsorption curve and a cavitation-induced *P*/*P*_0_ desorption branch at the lower *P*/*P*_0_ of its path.^46^ This indicates that mesoporous materials are plate-like aggregates and form a network of pores that remains partially filled by the pore condensate. Therefore, the pore shapes like cylindrical, slit, and macropores are involved. Indeed, particles like halloysite nanotubes and alike had a more clearance in the H3 loop than that for the kaolinite-rich materials (Figure S7); these particle arrangements are also seen in the SEM and TEM images (Figures 2, S4, and S5).

The isotherms generated in this study showed that all materials had some extent of gas sorption capacity at even a low relative pressure (*P*/*P*_0_ < 0.01), reflecting the role of micropores into this process (Figure S7).^47^ However, these materials did not end up with an adsorption plateau at the high relative pressure (*P*/*P*_0_ ∼ 1.0), resembling the type II adsorption branch of isotherms. An unrestricted monolayer–multilayer adsorption of gas up to high relative pressure characterizes these curves.^46^ Modification by acid or heat did not affect the high end of relative pressure-controlled gas adsorption. However, it increased this process in the micropores and the lower end of the mesopore region. This is a prominent feature of the iron-rich materials in which iron was leached (Figure S7).

In this study, an obvious difference was identified between the iron-rich clays and their iron-poor counterparts in terms of the pore-influenced desorption, where the former developed a prominent hysteresis loop. Wang et al.^47^ studied kaolinite and non-kaolinite clay minerals, such as illite and smectite and found that a visible loop developed in non-kaolin mineral particles. We also identified that the iron-rich variants of kaolin contain impurities of mica/illite (Figures 1 and 2).

## 4 Conclusions and Future Research

The presence of iron in kaolin minerals or as coexisting mineral species is commonly reported in raw clay deposits. However, the quantity, location and states of the iron are important chemical considerations. In the case of kaolin mined from the Australia Cloud Nine deposit, the iron was found to be predominantly nonstructural of the kaolin minerals and located in accessory mica or illite. Acid activation removed part of the iron, while heating was less effective. The surface charge, surface area, and pore volume patterns changed in relation to the kaolin mineral assemblages and the iron impurities. When iron was removed from the halloysite-associated impurities, these properties were enhanced and led to the adsorption of an increased number of gas molecules. As surface charges were also enhanced toward more negative charges by the acid activation, applications related to this property could usefully consider the distribution of iron. Future research is of interest for the usage of such kaolin deposits to discover whether removing iron or leaving it is required for target applications. Such applications include adsorption–desorption of gases and other pollutants or clay additive formulations for agrochemicals.

## Supporting Information Available

Drill and sampling locations; photographs of raw and modified clays; additional XRD, FTIR, and XRF results of studied samples; TEM and additional SEM images of samples; ζ-potential of all samples against range of pHs; and isotherm hysteresis curves for all samples (PDF)
