# Supplementary material for: Understanding Iron Impurities in Australian Kaolin and Their Effect on Acid and Heat Activation Processes of Clay
Source: ACS Omega. 2023 Feb 6;8(6):5533–44. doi: 10.1021/acsomega.2c06795 (PMC9933215; doi:10.1021/acsomega.2c06795)
Supplement: Supplementary file 1 — ao2c06795_si_001.pdf [file ao2c06795_si_001.pdf]

Supplementary information-

Understanding iron impurities in Australian kaolin and their effect on acid and heat activations processes of clay

Bhabananda Biswas[a,b,c*], Md. Rashidul Islam[a,b], Amal Kanti Deb[a,b], Anthony Greenaway[d], Laurence N. Warr[c], Ravi Naidu[a,b*]

[a] Global Centre for Environmental Remediation, The University of Newcastle, Callaghan, NSW 2308, Australia

[b] crcCARE Pty Ltd, ATC Building, the University of Newcastle, Callaghan, NSW 2308, Australia

[c]Institute of Geography and Geology, University of Greifswald, 17489 Greifswald, Germany
[d]Latin Resources Ltd, Unit 3, 32 Harrogate Street, West Leederville WA 6007, Australia

[*] Corresponding authors: email: Bhaba.Biswas@newcastle.edu.au (B. Biswas) and Ravi.Naidu@newcastle.edu.au (R. Naidu) p: +61 02 498 54501

SI 1. The drill location, sampling points and full list of supplied sample profiles

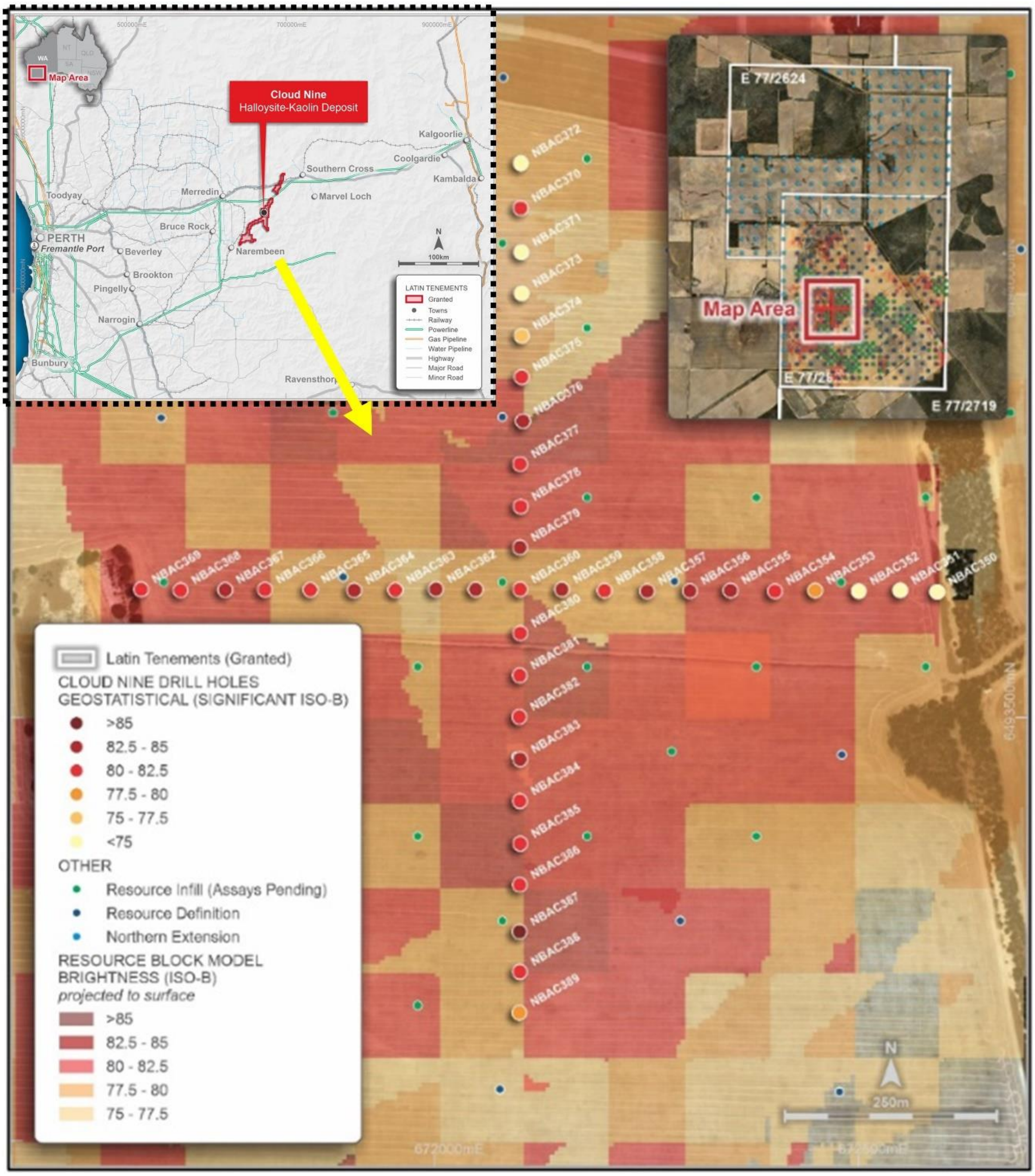

Figure S 1. *(Part A) The clay deposit and the drill location for extracting clay minerals. Figure is composed from the open access sketches, relating to the Cloud Nine Kaolin deposit, supplied by the Latin Resources Ltd.*

HoleID*	Depth From	DepthTo	Interval	Fe_2O_3 (%)	Al_2O_3 (%)	SiO_2(%)	TiO_2(%)	Kaolinite (%)	Halloysite (%)	Brightness (ISO-B)
NBAC353	32	34	2	5.71	26.2	51.6	1.79	39	28	38.5
NBAC354	39	40	1	5.29	27.2	50.6	1.97	31	39	41
NBAC354	38	39	1	3.44	28.3	51.49	2.31	40	32	48
NBAC356	44	45	1	0.97	33.2	50.93	0.46	56	23	77.5
NBAC356	42	44	2	0.88	34.1	49.63	0.36	63	19	79
NBAC356	36	38	2	0.77	32.7	50.92	0.41	34	40	80
NBAC356	38	40	2	0.76	34.2	50.05	0.37	42	37	79.5
NBAC356	34	36	2	0.59	33.5	50.85	0.25	43	32	83
NBAC357	42	44	2	1.01	33.8	50.88	0.31	59	19	79
NBAC357	38	40	2	0.8	34.1	50.21	0.32	52	29	81
NBAC357	40	42	2	0.73	33.9	50.53	0.34	51	29	81
NBAC357	34	36	2	0.61	33.3	50.82	0.32	41	35	80
NBAC357	32	34	2	0.54	33.4	51.32	0.27	55	20	80.5
NBAC358	28	30	2	0.32	34.3	50.36	0.45	20	58	82
NBAC358	30	32	2	0.32	34.7	49.82	0.48	43	39	80.5
NBAC358	9	10	1	1.77	29.8	55.71	0.95	61	19	48.5
NBAC358	36	38	2	0.94	34.2	50.29	0.4	57	25	74.5
NBAC358	14	16	2	0.58	37.6	48.59	0.37	70	26	77.5
NBAC358	20	22	2	0.5	37.6	47.53	0.37	73	23	84.5
NBAC358	26	28	2	0.47	33.7	50.93	0.38	36	40	82
NBAC358	34	36	2	0.41	34.7	50.26	0.29	40	41	83.5
NBAC358	32	34	2	0.4	34.6	50.11	0.4	42	38	82
NBAC358	18	20	2	0.36	37.8	47.06	0.39	50	47	85
NBAC360	18	20	2	0.47	35.3	49.69	0.5	64	19	80.5
NBAC360	16	18	2	0.43	34.2	50.45	0.58	60	20	80.5
NBAC360	14	16	2	0.43	35.1	49.54	0.54	64	19	80.5
NBAC361	10	12	2	0.57	35.1	50.67	0.09	68	19	85.5
NBAC361	14	16	2	0.54	34.2	50.78	0.11	59	20	85.5
NBAC361	12	14	2	0.54	36	49.01	0.08	64	24	87
NBAC364	19	21	2	1.3	33.9	50.21	0.22	50	29	63
NBAC365	20	21	1	0.68	34.1	50.28	0.22	44	36	73.5
NBAC365	19	20	1	0.6	36.2	48.34	0.31	62	28	82.5
NBAC365	17	19	2	0.58	37.6	47.58	0.3	70	26	84.5
NBAC365	15	17	2	0.56	37.8	47.27	0.29	72	25	85
NBAC365	13	15	2	0.48	38.2	47.16	0.28	74	24	86
NBAC366	28	30	2	0.43	33.5	50.76	0.48	28	47	80
NBAC367	36	38	2	0.41	32.9	51.43	0.32	53	21	83
NBAC367	30	32	2	0.39	34.3	50.24	0.43	58	21	82.5
NBAC367	26	28	2	0.32	37.9	47.2	0.38	72	23	85.5
NBAC367	34	36	2	0.3	33	51.19	0.37	48	26	84
NBAC367	28	30	2	0.28	36.1	48.73	0.42	61	26	84
NBAC368	17	19	2	0.58	37.3	44.91	1.23	74	20	77.5
NBAC368	21	22	1	0.54	37.5	46.43	0.71	67	29	77.5
NBAC368	19	21	2	0.48	37.7	46.95	0.43	46	50	81.5
NBAC368	13	15	2	0.44	38.1	47.02	0.13	69	28	85.5
NBAC368	24	26	2	0.37	34.4	50.58	0.29	51	29	85
NBAC368	22	24	2	0.34	36.3	47.87	0.45	64	27	82.5
NBAC369	9	11	2	0.38	37.5	47.49	0.46	76	20	83
NBAC369	21	23	2	0.49	35	49.31	0.45	59	25	81.5
NBAC369	11	13	2	0.42	37.7	47.11	0.4	76	20	82
NBAC369	25	27	2	0.4	33.6	50.43	0.46	50	26	82.5
NBAC369	19	21	2	0.4	36.5	48.09	0.4	59	30	82.5

HoleID*	Depth From	DepthTo	Interval	Fe_2O_3 (%)	Al_2O_3 (%)	SiO_2(%)	TiO_2(%)	Kaolinite (%)	Halloysite (%)	Brightness (ISO-B)
NBAC371	22	23	1	4.43	31.3	49.67	0.33	43	30	44
NBAC371	13	14	1	2.37	36.9	46.45	0.28	73	23	48
NBAC371	15	16	1	1.7	36.9	46.75	0.18	77	19	60
NBAC371	12	13	1	1.55	36.9	47.07	0.32	74	22	57
NBAC371	14	15	1	1.38	37.2	46.74	0.15	70	26	59.5
NBAC373	3	4	1	5.83	34.9	43.63	1	64	28	20.5
NBAC373	23	24	1	2.96	33.8	48.48	0.35	57	25	49
NBAC373	18	19	1	1.47	33.9	50.1	0.08	52	28	53.5
NBAC373	21	22	1	1.15	34.6	49.33	0.08	56	27	65.5
NBAC373	14	16	2	0.92	36.2	48.96	0.1	71	19	80.5
NBAC373	17	18	1	0.88	34.4	50.23	0.13	55	25	67
NBAC373	16	17	1	0.88	34.2	50.51	0.07	59	20	76
NBAC373	19	20	1	0.73	37	47.59	0.1	59	35	81.5
NBAC373	20	21	1	0.7	36.9	47.6	0.15	60	34	83
NBAC374	3	4	1	3.56	37.2	44.63	0.86	69	27	39
NBAC374	23	24	1	2.56	34.2	48.33	0.22	30	53	51.5
NBAC374	24	25	1	2.52	31	51.59	0.25	26	44	48
NBAC374	13	15	2	2.23	32.4	50.61	0.14	41	32	50.5
NBAC374	10	11	1	1.96	37.1	46.16	0.23	68	29	54.5
NBAC374	15	17	2	1.71	33.1	50.05	0.18	51	26	56.5
NBAC374	17	18	1	1.64	33.2	49.94	0.21	50	28	57.5
NBAC374	11	12	1	1.39	37.5	46.76	0.35	62	34	62
NBAC374	12	13	1	1.39	35.3	48.98	0.33	68	20	73
NBAC374	20	21	1	1.2	35.3	49.17	0.16	63	23	66.5
NBAC374	18	20	2	0.9	34.3	50.21	0.11	50	29	70
NBAC375	22	23	1	5.4	31.1	48.46	0.31	31	42	35
NBAC375	15	17	2	4.44	35.3	45.44	0.36	56	36	36.5
NBAC375	20	22	2	4.03	32.3	48.34	0.19	36	40	39
NBAC375	17	19	2	3.39	33	48.86	0.23	34	43	41.5
NBAC375	19	20	1	1.8	32.4	50.56	0.18	29	45	54.5
NBAC375	14	15	1	1.44	37	47.05	0.36	71	26	73
NBAC376	23	24	1	3.8	31.6	49.63	0.33	49	25	39
NBAC376	22	23	1	2.78	32.9	49.37	0.24	42	34	43
NBAC376	21	22	1	1.26	33.6	50.32	0.29	50	27	67.5
NBAC378	29	31	2	2.45	34.9	47.93	0.19	68	20	52.5
NBAC378	7	9	2	1.11	32.2	53.88	0.3	67	20	68
NBAC378	9	11	2	0.57	35.8	49.19	0.16	64	24	83
NBAC379	21	23	2	0.61	35.2	49.33	0.31	67	19	83.5
NBAC379	19	21	2	0.41	35.2	49.52	0.28	62	22	85
NBAC380	14	16	2	0.41	37.7	47.01	0.41	78	19	83.5
NBAC380	20	22	2	0.4	34.8	49.61	0.56	55	27	80
NBAC380	26	28	2	0.36	35.7	49.22	0.48	59	28	83.5
NBAC380	16	18	2	0.36	37.2	47.97	0.41	63	30	83
NBAC381	29	31	2	0.53	35.3	49.39	0.56	66	19	79.5
NBAC381	23	25	2	0.45	34.3	50.25	0.59	60	20	79
NBAC382	14	16	2	0.57	38	47.43	0.39	72	25	84
NBAC382	18	20	2	0.49	38	46.94	0.42	69	27	82.5
NBAC383	11	13	2	0.29	38.2	47.27	0.33	77	21	83

*Highlighted one used in this study

color code	Key group/mineral
(blue)	Iron oxides
(orange)	Kaolinite
(green)	Halloysite

Figure S1. *(Part B) The dill profiles of the studied region. The mineral composition was obtained by independent consultation.*

SI 2. The physical appearance of sample clays

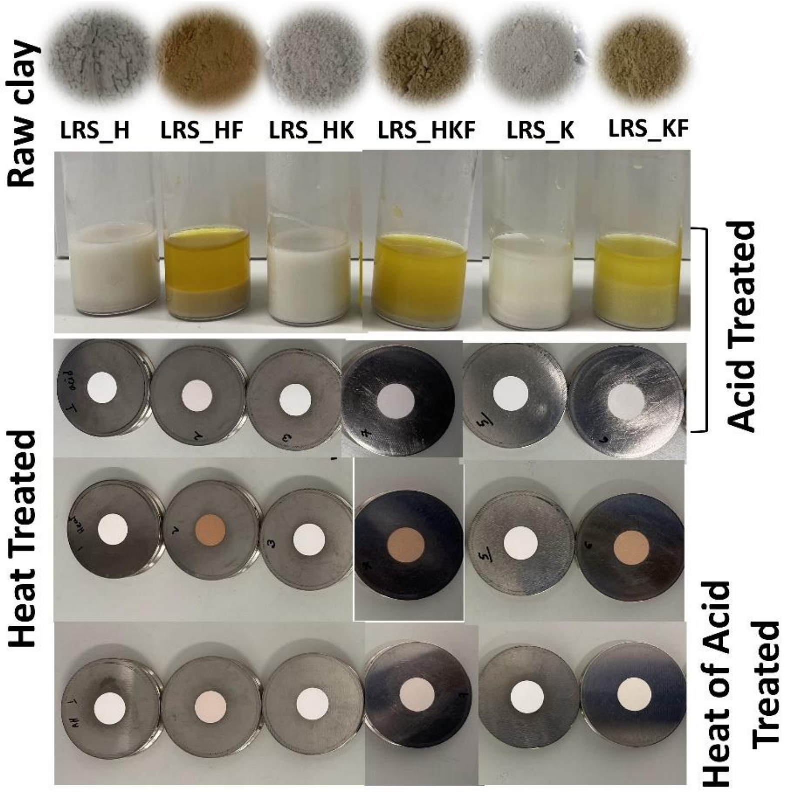

Figure S 2. *The physical look of the raw and modified clays. Hint: LRS denotes Latin Resources Ltd (the industry). LRS_H = industry supplied halloysite bulk, and LRS_HF = industry supplied iron rich halloysite bulk. Likewise, HK = halloysite-kaolinite, and HKF = iron rich variant of HK; K = kaolinite, and KF = iron rich variant of kaolinite.*

SI 3. Additional mineral and impurities characteristics

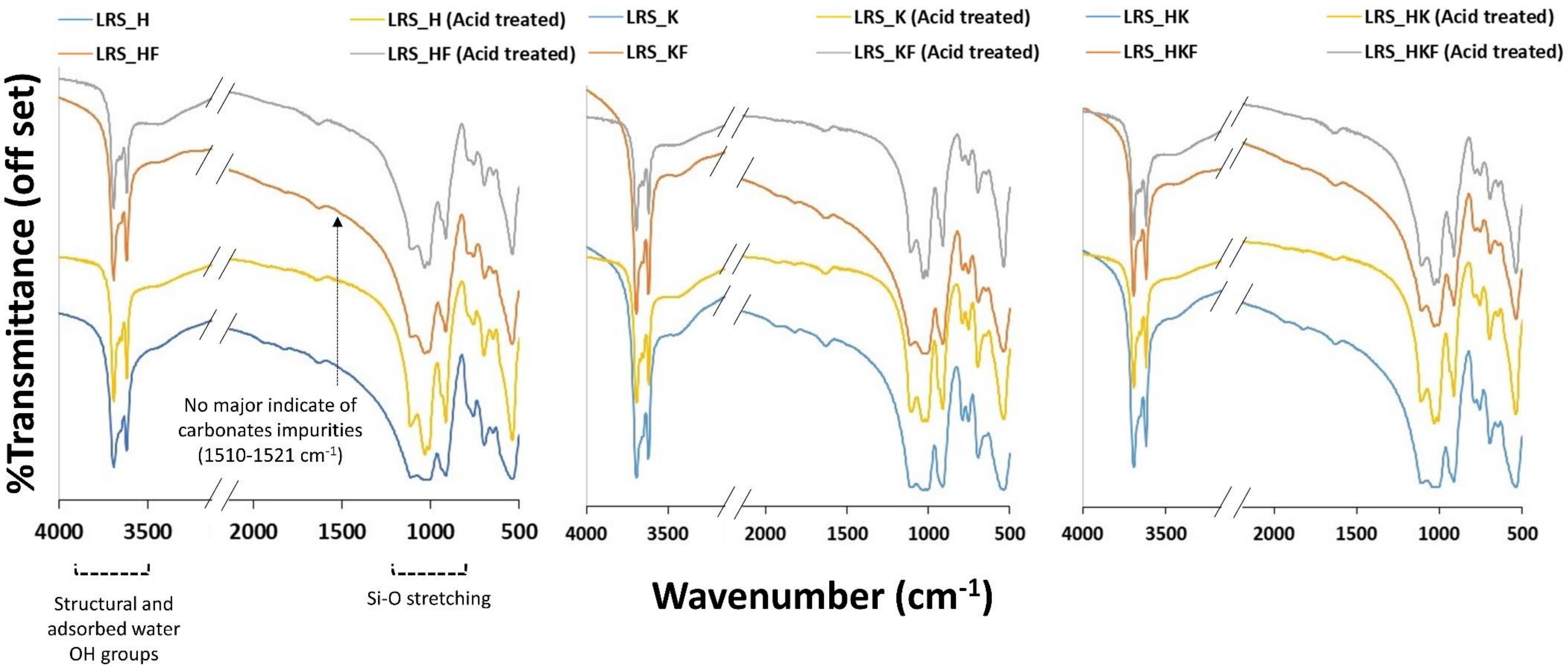

Figure S 3. The FTIR spectra of raw and selective modified clay samples.

SI 4. XRD pattern of the clay and modified clays

Table S 1. *Mineral, inter-planar spacing and relative intensity. The full pattern is presented in the main text*

	**Kaolin: d-spacing d_{001} (Å) at 2θ = 12.4°** [Relative Intensity, %]				**Mica/Illite: d-spacing d_{001} (Å) at 2θ = 8.8°** [Relative Intensity, %]			
**Samples**	**Raw**	**Acid**	**Heat**	**A.Heat**	**Raw**	**Acid**	**Heat**	**A.Heat**
LRS_H*	7.18 [100]	7.19 [100]	NA	NA	NA	NA	NA	NA
LRS_HF	7.16 [67.29]	7.17 [45.03]	NA	NA	10.01 [39.30]	10.00 [2.34]	9.98 [31.69]	NA
LRS_HK	7.19 [100]	7.19 [100]	NA	NA	NA	NA	NA	NA
LRS_HKF	7.14 [100]	7.18 [100]	NA	NA	9.99 [34.33]	10.05 [4.94]	10.02 [49.80]	10.04 [5.57]
LRS_K*	7.17 [100]	7.18 [100]	NA	NA	NA	NA	NA	NA
LRS_KF	7.17 [100]	7.18 [100]	NA	NA	10.04 [16.05]	10.04 [3.37]	9.99 [21.96]	NA

** Crystallite size: LRS_H = ~37 nm, and LRS_K = ~24 nm. Crystallite size (nm) was measured as Scherrer crystallite size using HighScore Plus software. Machine error used was 0.008 °2θ while the peak position and FWHM value were obtained from the "Peak Label" option offered in the HighScore software. NA = not available; Relative intensity measured using the HighScore Plus software [see details in Materials & Methods section of the main text].*

SI 5. XRF profile of raw and modified clays

Table S 2. *The XRF profile of the major oxides before and after the activations*

Com. (%)	LRS_H				LRS_HF			
	**Raw**	**Heat**	**Acid**	**A.Heat**	**Raw**	**Heat**	**Acid**	**A.Heat**
Al_2O_3	36.19	38.57	36.62	37.98	29.80	32.80	31.09	31.60
SiO_2	55.73	54.03	55.27	54.81	56.48	55.52	61.38	61.34
Si/Al	1.54	1.40	1.51	1.44	1.90	1.69	1.97	1.94
Fe_2O_3	0.56	0.52	0.51	0.45	6.07	5.22	1.48	1.34
TiO_2	0.46	0.43	0.48	0.43	0.47	0.37	0.27	0.25
K_2O	5.96	5.59	6.22	5.64	4.68	4.10	3.94	3.76
**Com. (%)**	**LRS_HK**				**LRS_HKF**			
	**Raw**	**Heat**	**Acid**	**A.Heat**	**Raw**	**Heat**	**Acid**	**A.Heat**
Al_2O_3	38.55	40.34	39.07	40.18	30.08	31.52	30.49	31.37
SiO_2	54.71	53.89	54.62	54.24	56.78	56.34	61.09	60.85
Si/Al	1.42	1.34	1.40	1.35	1.89	1.79	2.00	1.94
Fe_2O_3	0.40	0.34	0.30	0.26	5.33	4.84	1.61	1.50
TiO_2	0.66	0.57	0.64	0.56	2.00	1.89	2.04	1.87
K_2O	4.72	4.07	4.53	4.08	4.30	4.04	3.63	3.35
**Com. (%)**	**LRS_K**				**LRS_KF**			
	**Raw**	**Heat**	**Acid**	**A.Heat**	**Raw**	**Heat**	**Acid**	**A.Heat**
Al_2O_3	40.53	45.45	43.79	45.16	36.90	39.78	37.92	39.89
SiO_2	56.78	52.47	53.94	52.85	54.41	52.86	56.60	54.89
Si/Al	1.40	1.15	1.23	1.17	1.47	1.33	1.49	1.38
Fe_2O_3	0.57	0.45	0.46	0.42	3.74	3.24	1.34	1.32
TiO_2	0.67	0.54	0.59	0.54	0.32	0.27	0.26	0.25
K_2O	0.38	0.31	0.30	0.28	3.59	3.03	3.07	2.98

SI 6. TEM images of raw and acid-treated halloysite and kaolinite & SEM images

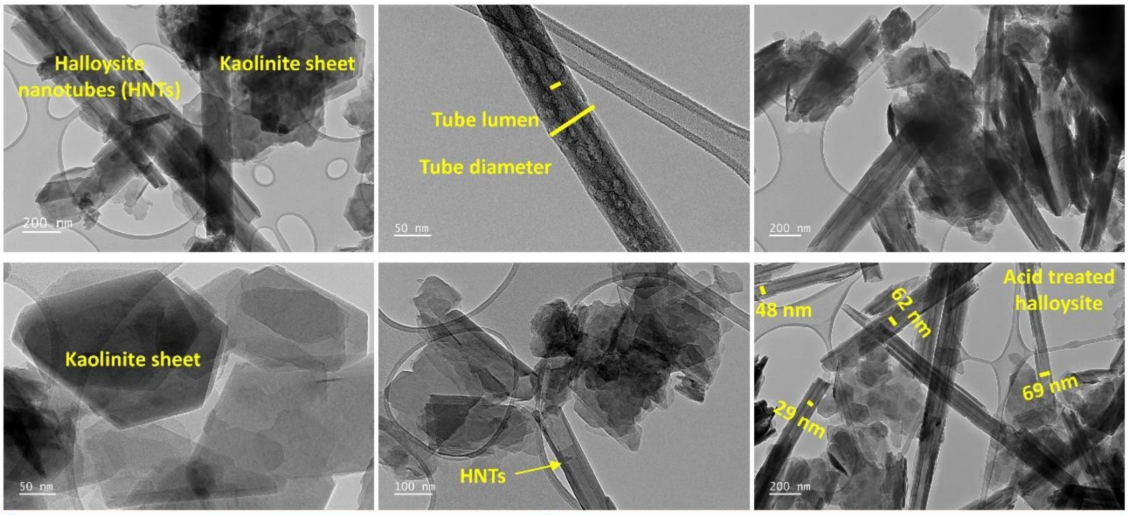

Dimension (Hal)	nm
Tube length	1035.6 ± 606.3
Tube diameter	153.9 ± 41.9
Lumen diameter	40.7 ± 12.9
Lumen diameter after acid	52 ± 17.0; max = 69, min = 31.0; med = 49.0.

Figure S 4. *The random glimpse of halloysite nanotube and kaolinite sheet under TEM. The length and diameter statistics were generated from the original SEM and TEM images using imageJ software.*

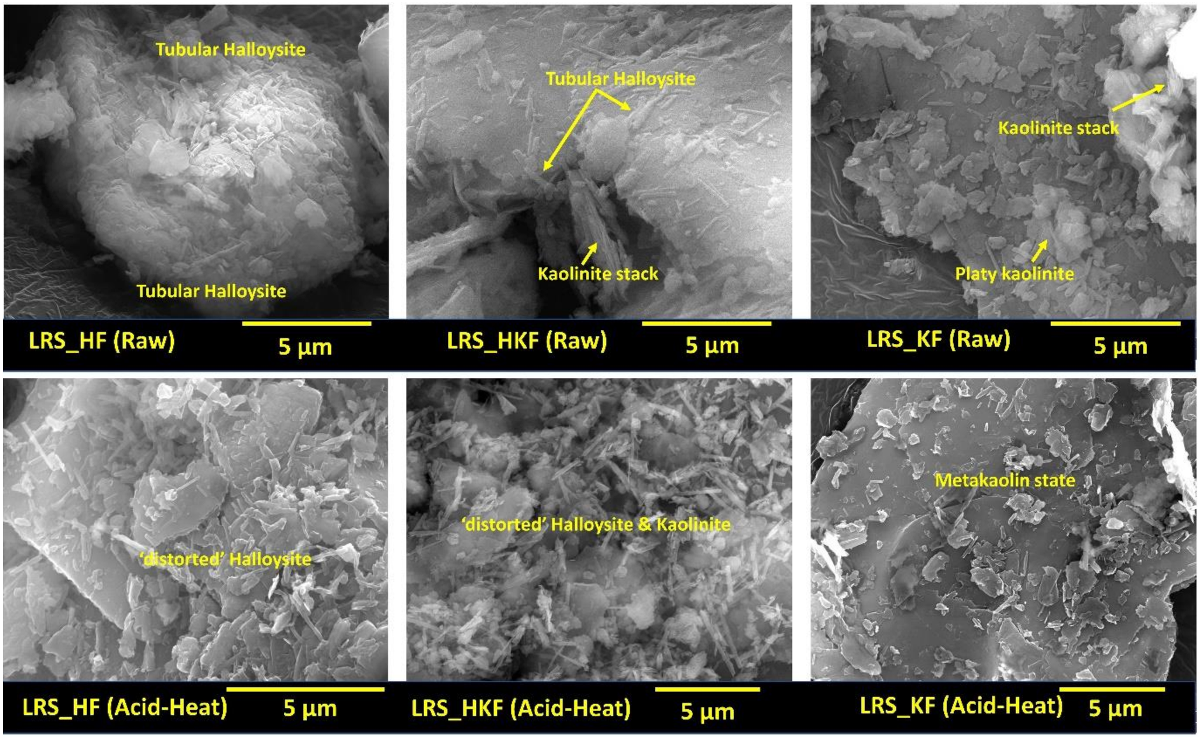

Figure S 5. *SEM images of Acid-Heat treated kaolin.*

SI 7. Zeta potential values against ranges of pHs and gas adsorption-desorption isotherm curve.

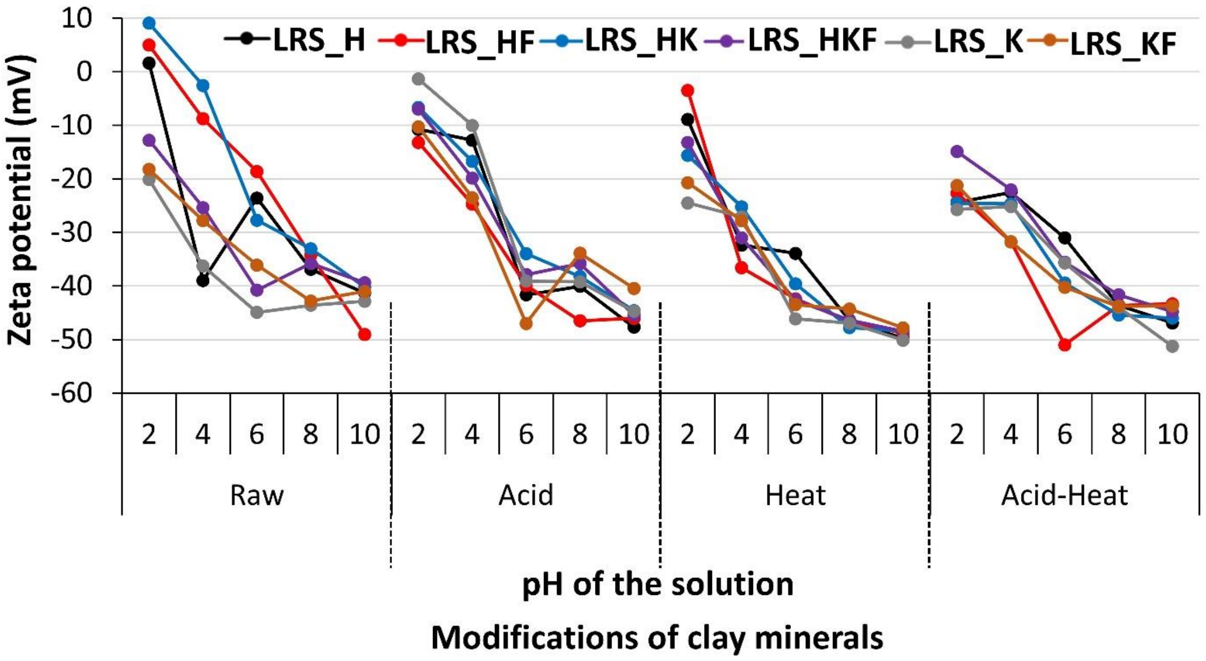

Figure S 6. *Zeta potential of clays and modified clays against various solution pHs.*

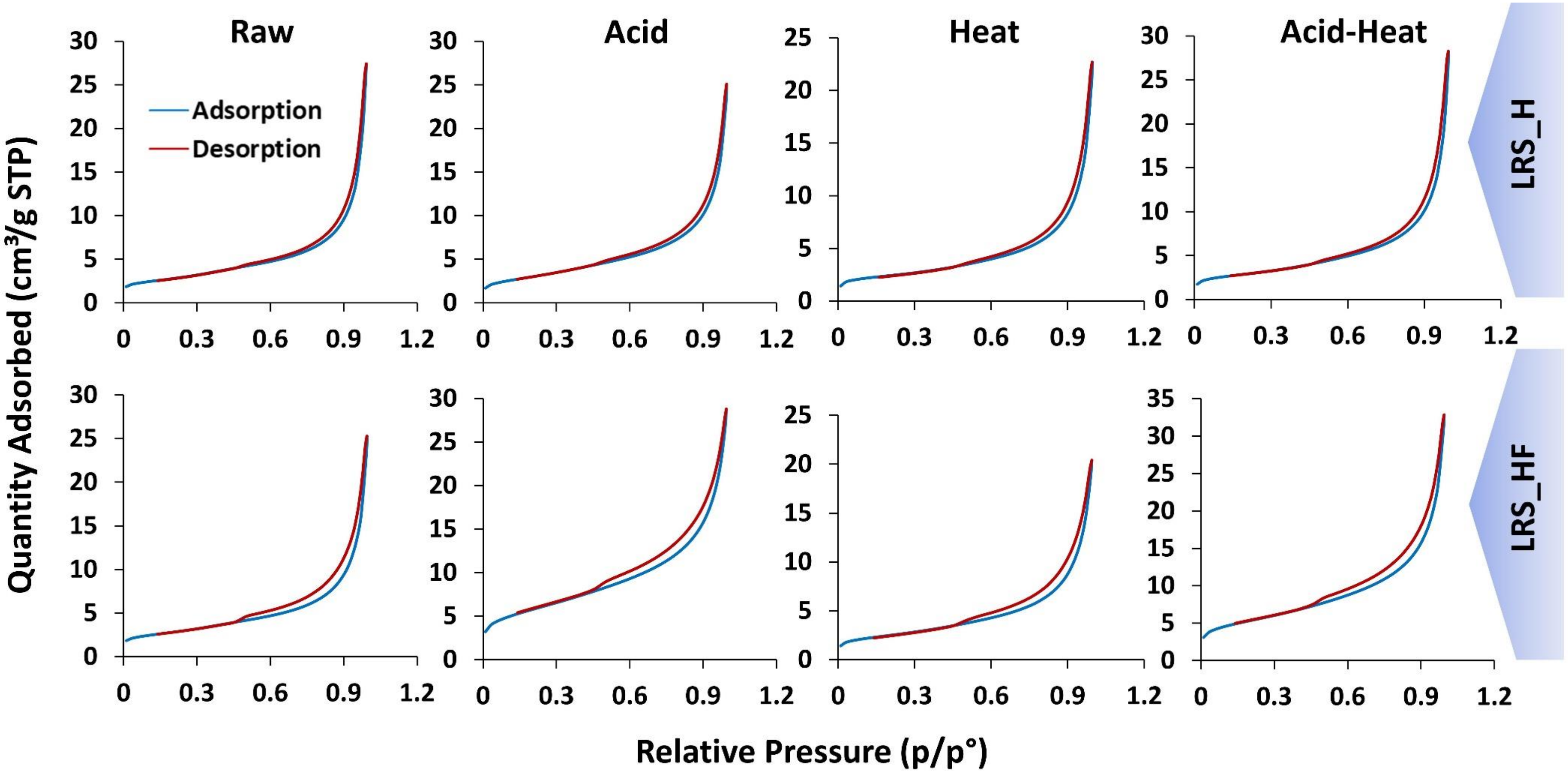

Figure S 7. *(Part A) The adsorption isotherm hysteresis of raw and treated LRS_H and LRS_HF. Hint: LRS_H = industry supplied halloysite bulk, and LRS_HF = industry supplied iron rich halloysite bulk.*

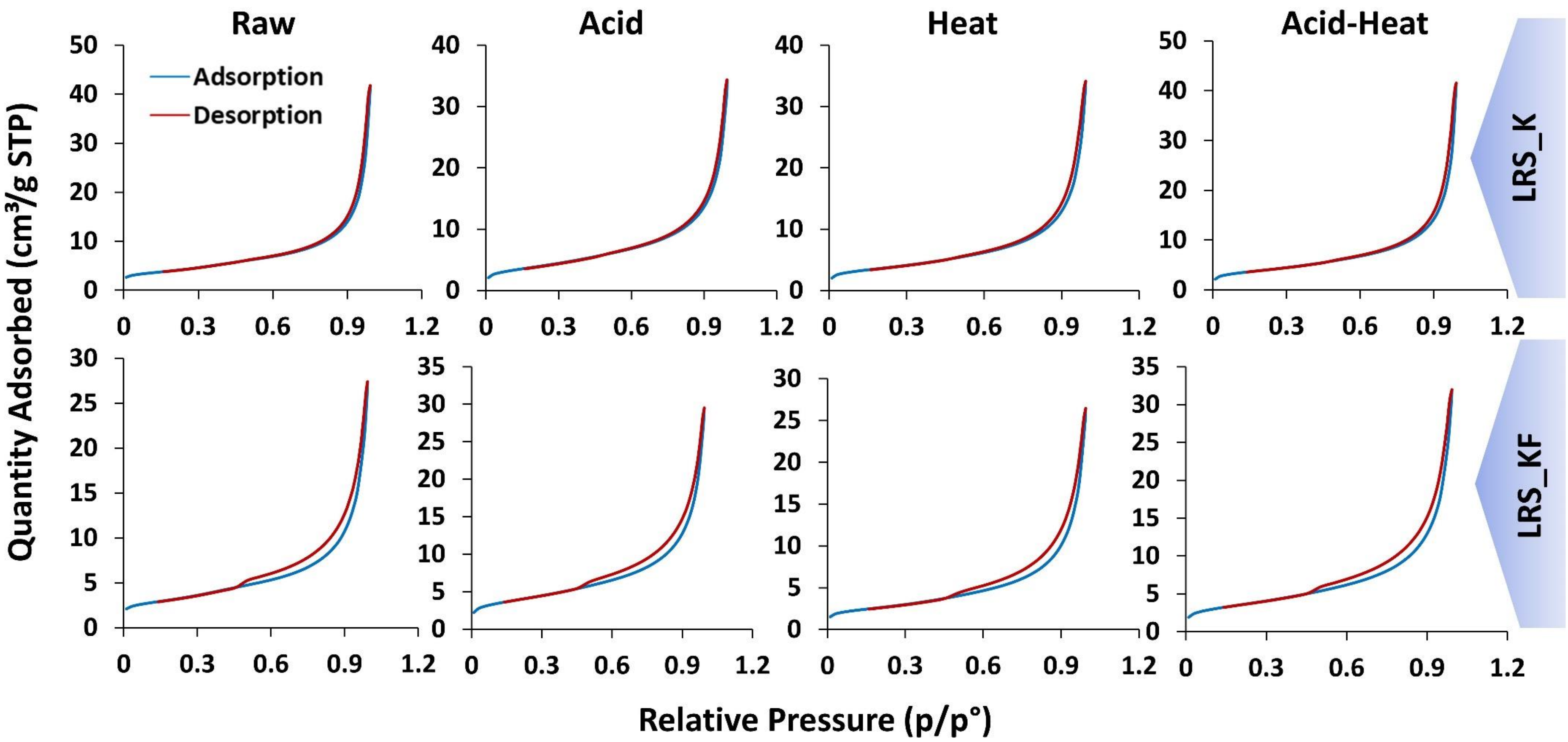

Figure S 7. *(Part B) The adsorption isotherm hysteresis of raw and treated LRS_K and LRS_KF. Hint: LRS_K = industry supplied halloysite bulk, and LRS_KF = industry supplied iron rich halloysite bulk.*

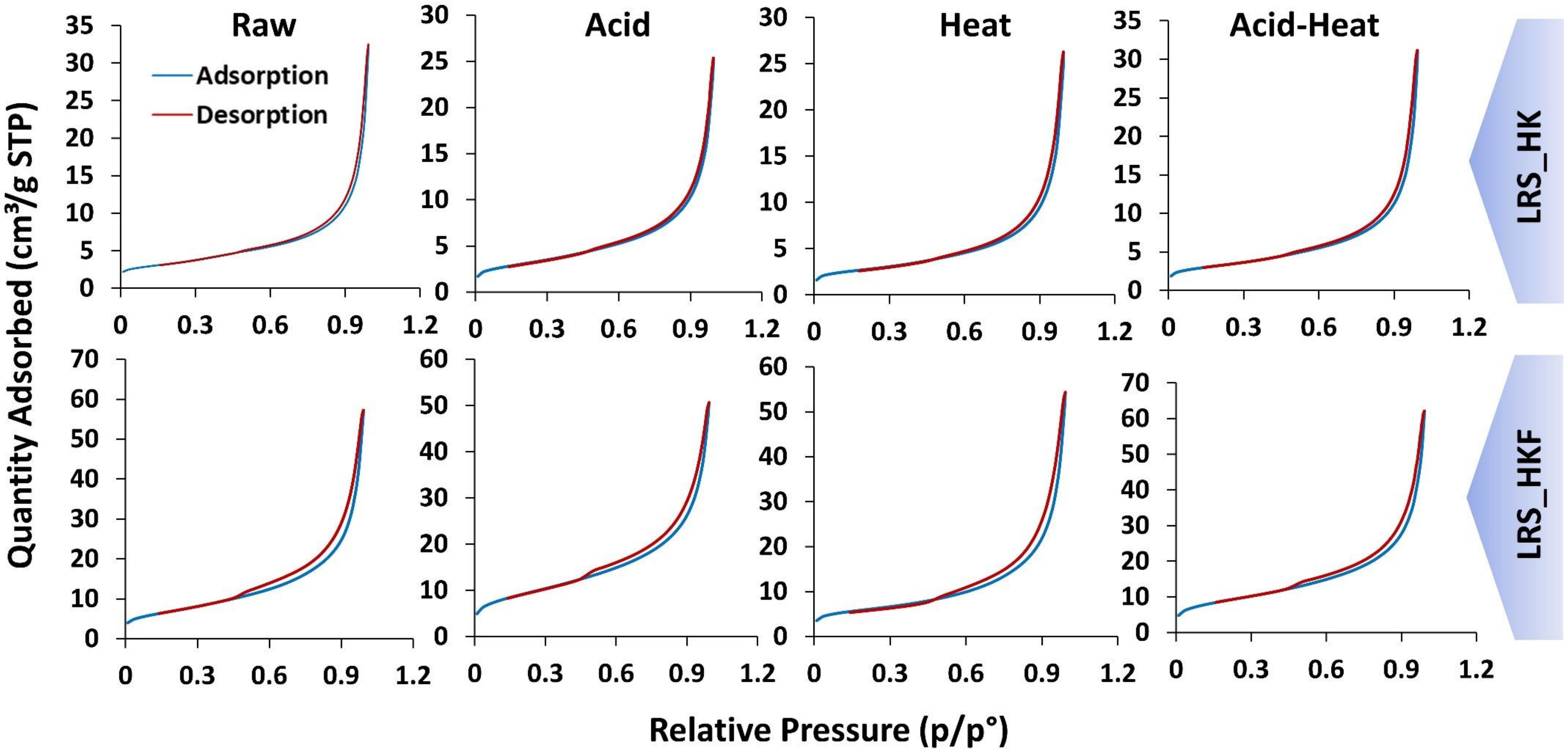

Figure S 7. *(Part C) The adsorption isotherm hysteresis of raw and treated LRS_HK and LRS_HKF.*
